# Efficient Recovery of Complete Gut Viral Genomes by Combined Short‐ and Long‐Read Sequencing

**DOI:** 10.1002/advs.202305818

**Published:** 2024-01-19

**Authors:** Jingchao Chen, Chuqing Sun, Yanqi Dong, Menglu Jin, Senying Lai, Longhao Jia, Xueyang Zhao, Huarui Wang, Na L. Gao, Peer Bork, Zhi Liu, Wei‐Hua Chen, Xing‐Ming Zhao

**Affiliations:** ^1^ Key Laboratory of Molecular Biophysics of the Ministry of Education Hubei Key Laboratory of Bioinformatics and Molecular Imaging Center for Artificial Intelligence Biology Department of Bioinformatics and Systems Biology College of Life Science and Technology Huazhong University of Science and Technology Wuhan Hubei 430074 China; ^2^ Department of Neurology Zhongshan Hospital and Institute of Science and Technology for Brain‐Inspired Intelligence Fudan University Shanghai 200433 China; ^3^ College of Life Science Henan Normal University Xinxiang Henan 453007 China; ^4^ Department of Laboratory Medicine Zhongnan Hospital of Wuhan University Wuhan University Wuhan 430071 China; ^5^ European Molecular Biology Laboratory Structural and Computational Biology Unit 69117 Heidelberg Germany; ^6^ Max Delbrück Centre for Molecular Medicine 13125 Berlin Germany; ^7^ Yonsei Frontier Lab (YFL) Yonsei University 03722 Seoul South Korea; ^8^ Department of Bioinformatics Biocenter University of Würzburg 97070 Würzburg Germany; ^9^ Department of Biotechnology College of Life Science and Technology Huazhong University of Science and Technology 430074 Wuhan China; ^10^ Institution of Medical Artificial Intelligence Binzhou Medical University Yantai 264003 China; ^11^ MOE Key Laboratory of Computational Neuroscience and Brain‐Inspired Intelligence and MOE Frontiers Center for Brain Science Fudan University Shanghai 200433 China; ^12^ State Key Laboratory of Medical Neurobiology Institute of Brain Science Fudan University Shanghai 200433 China; ^13^ International Human Phenome Institutes (Shanghai) Shanghai 200433 China

**Keywords:** crAssphage, gubaphage, gut virome, long‐read sequencing, pacBio sequel II, terminase, virus‐like particle

## Abstract

Current metagenome assembled human gut phage catalogs contained mostly fragmented genomes. Here, comprehensive gut virome detection procedure is developed involving virus‐like particle (VLP) enrichment from ≈500 g feces and combined sequencing of short‐ and long‐read. Applied to 135 samples, a Chinese Gut Virome Catalog (CHGV) is assembled consisting of 21,499 non‐redundant viral operational taxonomic units (vOTUs) that are significantly longer than those obtained by short‐read sequencing and contained ≈35% (7675) complete genomes, which is ≈nine times more than those in the Gut Virome Database (GVD, ≈4%, 1,443). Interestingly, the majority (≈60%, 13,356) of the CHGV vOTUs are obtained by either long‐read or hybrid assemblies, with little overlap with those assembled from only the short‐read data. With this dataset, vast diversity of the gut virome is elucidated, including the identification of 32% (6,962) novel vOTUs compare to public gut virome databases, dozens of phages that are more prevalent than the crAssphages and/or Gubaphages, and several viral clades that are more diverse than the two. Finally, the functional capacities are also characterized of the CHGV encoded proteins and constructed a viral‐host interaction network to facilitate future research and applications.

## Graphical abstract

The CHGV, a human gut virome database, comprises 21,499 non‐redundant phage genomes obtaine through viral‐like particle enrichment and combined short‐ and long‐read sequencing. The long‐reads facilitate the identification of more complete viral genomes and surprisingly favor lowly abundant ones. The CHGV features novel genomes, prevalent phages exceeding crAssphages/Gubaphages, and enables functional analysis and viral‐host interaction network construction. This enhances gut virome research and applications significantly.

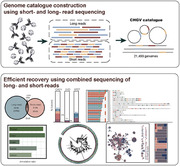

## 1 Introduction

The gut viral community (also known as the gut virome), mainly consisting of bacteriophages and archaeal viruses, has been shown to be diverse in the human gut.^[^ ^1^ ^]^ Viruses play crucial roles in shaping the gut microbial composition and hold great promise for the precision manipulation of the gut bacteriome. Despite tremendous advancements in identifying human (gut) viral genomes,^[^ ^2^ ^]^ the gut virome has been far less well characterized than the prokaryotic community.^[^ ^3^ ^]^ Most importantly, the diversity of the gut virome has been vastly underestimated because of biological and technical challenges.^[^ ^4^ ^]^

There are two main approaches for viral sequencing from metagenomes: whole microbial community sequencing (metagenomics)^[^ ^2c^ ^]^ and VLP sequencing. The first approach involves direct identification of viral contigs assembled from metagenomic datasets with the help of viral‐detection bioinformatics tools.^[^ ^5^ ^]^ Recently, several large human viral genome catalogs have been established in this way, including the Gut Phage Database (GPD),^[^ ^2b^ ^]^ Metagenomic Gut Virus catalog (MGV),^[^ ^2c^ ^]^ Cenote‐Taker 2–compiled Human Virome Database (CHVD),^[^ ^2d^ ^]^ and database for extrachromosomal mobile genetic elements (mMGE).^[^ ^2f^ ^]^ However, these datasets are biased towards the highly abundant viruses and against uncharacterized ones. Another approach is the VLP sequencing, which usually involves the removal of human and bacterial cells and DNAs, followed by virus concentration. This enriches for viral particles and can reveal less abundant or novel viruses missed by metagenomic sequencing. Representative databases that use this approach include the GVD^[^ ^6^ ^]^ and Danish Enteric Virome Catalog (DEVoC).^[^ ^2e^ ^]^ Although human feces contain much greater numbers of VLPs than environmental samples,^[^ ^7^ ^]^ they are rich in organic solids as compared with environmental samples,^[^ ^7a^ ^]^ which can greatly impede VLP purification. Most previous studies thus have used whole‐genome amplification methods, such as multiple displacement amplification (MDA)^[^ ^8^ ^]^ to obtain sufficient amounts of DNA for sequencing.^[^ ^8^, ^9^ ^]^ However, the MDA method has been known to suffer from significant drawbacks including uneven genome coverage, chimeric sequences, and biased amplification.^[^ ^10^ ^]^ A few recent studies explored amplification‐independent approaches to obtain VLP sequencing data via direct VLP purification from human feces but included very few samples (≤ 30).^[^ ^11^ ^]^ Third‐generation sequencing technologies, including Nanopore and PacBio technology, could also be applied to VLP sequencing to generate longer viral reads; however, as they require even higher‐quality viral DNA in larger quantities, these methods have been applied to even fewer samples (e.g., ≤ 10).^[^ ^12^ ^]^

Here, we report a comprehensive virus detection procedure for the human gut virome involving the VLP enrichment from an increased amount of feces (≈500 g per person), compared to previous studies (0.5–5 g per person),^[^ ^13^ ^]^ combined Illumina and PacBio sequencing, and comprehensive bioinformatics analysis. When applied it to fecal samples from 180 healthy Chinese individuals, we constructed a collection of 21499 non‐redundant vOTUs via integrated assembly of the short and long reads. The availability of both Illumina and Pacbio sequencing has allowed us to further evaluate the advantages of long‐read sequencing in assembling of viral genomes. For example, the vOTUs assembled from PacBio long reads were longer and included a higher proportion of complete genomes compared to Illumina short‐read assemblies. Approximately 37% (5017) of the PacBio assemblies were complete genomes, versus 30% (2437) for Illumina. Furthermore, we found that both short‐read group and long‐read group contained unique sets of vOTUs, while long‐reads demonstrates a greater ability to detect viral taxa that were present in low abundance within our cohort or were recognized to possess large genomes. With long‐read involved, we estimated that 35% of the total vOTUs were complete and 41% were high‐quality (i.e., included the completed ones), which was significantly higher than that in the GVD (≈4%; 6%) and GPD (12%; 29%).

This viral dataset, referred to as the CHGV collection, extends our knowledge of the human gut virome from several aspects. For example, we identified several viruses that are more prevalent than crAssphages and Gubaphages, the two most diverse gut viral clades known in the human gut microbiome so far.^[^ ^2b^ ^]^ In addition, we revealed key features of the human gut virome such as that it was dominated by virulent viruses that are more diverse, prevalent, and abundant, although the majority of the gut phages were temperate. We also assigned ≈35% of the virus with their bacteria hosts. In summary, by combining short‐ and long‐read sequencing, we reveal the hidden diversity of the gut virome using combined short‐ and long‐read sequencing and broaden our knowledge of viral dark matter in human gut microbial ecology.

## 2 Results

### 2.1 Combined Short‐ and Long‐Read Sequencing is Highly Efficient in Recovering Longer and More Complete Gut Phage Genomes

**Figure 1:**
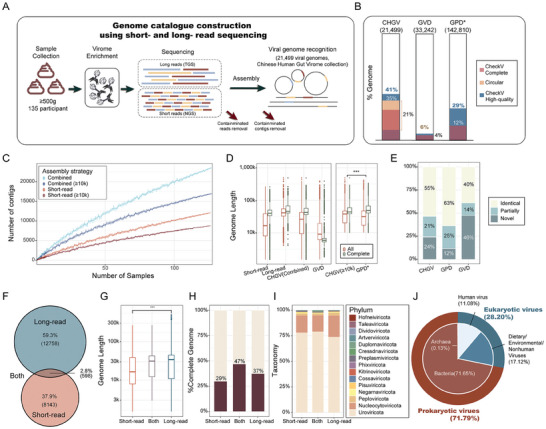
Efficient recovery of high‐quality gut vOTUs using combined sequencing of long‐ and short‐reads. A) Combined assembly of long‐ and short‐read generated aCHGV collection containing ≈35% complete phage genomes. B) Bar plot comparing the complete genome ratio among databases (Dark red: CheckV^[^ ^22^ ^]^ completeness 100%, ≈28%; light pink: circular genome, ≈21%; Dark blue: CheckV high‐quality, ≈41%). GVD: The Gut Virome Database; GPD: the Gut Phage Database. * Note the GPD catalogue only included phage genomes >10k. C) Rarefaction curves of non‐redundant/unique phage contigs obtained from the short‐read and combined‐assemblies. D) Genome length comparisons of the vOTUs obtained by the different assemblies and in selected public viral catalogues. Our CHGV vOTUs, when limiting to those of ≥  10K (the same criterium used in GPD), are significantly longer than those in the GPD database(Wilcoxon rank sum test, p<0.01); although the complete genomes in CHGV are shorter. Here, the long‐read and short‐read represents the assembly methods. E, Bar plot showing the novelty of the CHGV and selected public human viral catalogues as compared with all other human viral catalogues including GVD, GPD, CHVD,^[^ ^19^ ^]^ DEVoC,^[^ ^2e^ ^]^ and MGV.^[^ ^2c^ ^]^ Identical: ≥ 95% ANI; partially: ≥ 70% ANI; novel <70% ANI. F) Venn diagram showing the contributions of the long‐ and short‐read to the CHGV vOTUs. The criteria for assigning the vOTUs into the three groups, namely Long‐read, Both and Short‐read were shown in Figure S10 (Supporting Information). Briefly, a virus will be assigned to the Long‐read (Short‐read) group if it can only be assembled using the long(short)‐read. G,H,I) Genome length distribution (G), ratios of complete genomes (H), and taxonomic annotation (I) of the three groups. J) Host distribution of the vOTUs based on the host information from the Virus‐Host DB^[^ ^23^ ^]^; the host assignment was performed at the family level annotation.

To survey the human gut virome without being limited by the known restrictions of previous methods, we applied a comprehensive VLP enrichment protocol to fecal samples (≈500 g each) from 135 healthy Chinese participants. This allows us to extract large quantities of high‐quality, high‐molecular‐weight DNA from dsDNA viruses (Experimental Section). We subjected all qualified samples to viral Illumina short‐read sequencing and those with enough DNA to PacBio long‐read sequencing (Figure 1A; Experimental Section). We also performed to regular whole‐microbial community sequencing (metagenomic next‐generation sequencing, mNGS) on all fecal samples. In total, we obtained 135 viral short‐read sequencing datasets, 83 viral long‐read sequencing datasets, and 135 mNGS qualified sequencing datasets (Table S1, Supporting Information).

Following the elimination of human host and bacterial contaminants, we executed a comprehensive assembly process on the resultant clean reads using an integrated approach, combining viral short‐read, long‐read, and hybrid assemblies (Experimental Section). These assemblies were de‐replicated based on an average nucleotide identity (ANI) threshold of 95% global sequence identity, resulting in a 100% sequence consistency for 95% of the shorter genome. This yielded a total of 97513 non‐redundant contigs that met the criteria of being≥5 kb in length. Our selection process for contigs involved employing six widely‐recognized viral detection pipelines (Methods), including VirSorter,^[^ ^14^ ^]^ VirFinder,^[^ ^15^ ^]^ and PPR‐Meta,^[^ ^16^ ^]^ along with the nucleotide sequence similarity searches using BLAST, searches for phage homologous proteins also via BLAST and the evaluation of contig completeness with CheckV.^[^ ^17^ ^]^ Specifically, we retained contigs that were either identified as viral by at least two detection pipelines (18739), or identified by a single pipeline while also considered as “high‐quality” according to CheckV (2790). In addition, we excluded vOTUs exhibiting 90% sequence similarity to UHGG‐minus (the UHGG catalogue with prophage sequences removed; see Experimental Section) spanning at least 50% of the total length (resulting in the removal of 1867 vOTUs). Through careful screening and quality control, we compiled a catalog comprising 21499 vOTUs, which we have designated as theCHGV catalog (Figure 1A; Table S2, Supporting Information). It is important to clarify that the term “Chinese” is employed to denote the origin of the samples and does not imply that the CHGV vOTUs are necessarily representative of the broader Chinese population. Notably, it was observed that longer contigs exhibited a higher probability of recognition within our pipeline, as well as by individual viral identification tools (Figure S1, Supporting Information).

A substantial 34.69% (7510) of CHGV vOTUs achieved completeness either through CheckV (5659 phages) or circularity (4586, Experimental Section; see ref.[18]). This is 7–10 times higher than GVD^[^ ^6^ ^]^ (4%), 3–4 times increase (under the same length filtering criteria, i.e., > 10 kb; ≈42%) comparing to GPD^[^ ^2b^ ^]^ (12%)(Figure 1B),11‐12 times increase than comparing to mMGE and 16–17 times increase than comparing to CHVD (Figure S2, Supporting Information). Our combined assembly yielded more diverse vOTUs per sample than short‐read alone (Figure 1C) and longer vOTUs than GVD,mMGE, DEVoC (and GPD under the same criteria, Figure 1D). However, vOTUs in CHGV did not exhibit longer genome length over MGV and CHVD, neither in terms of overall vOTUs nor specifically for fragments exceeding 5 kb (Figure S3, Supporting Information), this could potentially be attributed to the diverse sample sources of CHVD and the substantial sample sizes included in both MGV and CHVD. Notably, our CHGV catalog contained 32% novel vOTUs (ANI <70% with public viruses; Experimental Section), absent in published datasets like GVD,^[^ ^6^ ^]^ GPD,^[^ ^2b^ ^]^ CHVD,^[^ ^19^ ^]^ DEVoC,^[^ ^2e^ ^]^ and MGV,^[^ ^2c^ ^]^ significantly exceeding GPD's (≈12% novel vOTUs), MGV(≈3.8% novel vOTUs, Figure S4, Supporting Information). GVD had 46% novel phages (Figure 1E) due to its larger sample size (2697 VLP‐samples). This trend toward the discovery of novel vOTUs is also evident in the CHVD and mMGE datasets. This may be due to their broader range of sample origins, extending beyond just the enteric category, which includes a significant number of novel vOTUs (Figure S4, Supporting Information).Our combined assembly of long‐ and short‐read produced longer viral vOTUs in CHGV, with more complete genomes than public databases and a substantial portion of novel ones.

### 2.2 Combined Sequencing Identifies Significantly More Gut Viruses Than Short‐Reads

To assess the impact of long‐read sequencing on the construction of CHGV vOTUs, we categorized the 21499 vOTUs into three distinct groups based on the necessity of long‐read during their assembly process. This categorization is depicted in Figure S5 (Supporting Information) and involves the following criteria: 1) A virus is assigned to the Long‐read group if it was assembled using either long‐read or hybrid (a combination of long‐ and short‐reads) assembly methods, and none of the short‐read assembled contigs that were excluded during de‐replication shared over 95% ANI with it, while also covering more than 50% of its total length. This implies that such a virus was exclusively assembled with the assistance of long‐read. 2) Viruses are assigned to the Short‐read group if they were exclusively assembled using short‐read. 3) Viruses that could be assembled using both short‐read and long‐read methods were placed in the Both group. As shown in Figure 1F, our analysis indicated that the Long‐read group encompassed 59.8% of the CHGV vOTUs, including vOTUs assembled exclusively by long‐read assembled methods (15.9%), and those assembled only by hybrid assembled methods (42.7%; Figure S6, Supporting Information).The Short‐read group represents a smaller portion(37.4%), and an even lesser number of vOTUs, ≈2.8%, are categorized under Both group (Figure 1F). Notably, when we limited our examination to samples with both viral short‐read and viral long‐read sequencing data, similar outcomes were observed (Figure S7, Supporting Information). These findings strongly suggest that the majority of assembled vOTUs benefited significantly from the incorporation of long‐read sequencing techniques.

We then proceeded to delve into the characteristics of the vOTUs underlying the aforementioned classifications. We first categorized all genomes based on their assembly methods and found that assemblies involving long reads consistently yielded longer vOTUs, as well as a higher proportion of complete vOTUs. This indicates that the inclusion of long reads during assembly enhances the length and completeness of vOTUs (Figures S8 and S9, Supporting Information). We also assessed the prevalence of vOTUs from different sources and found that vOTUs in the Both group are widely distributed, which may make them easily detectable (Figure S10, Supporting Information). Surprisingly, we noted a notably lower abundance of long‐read vOTUs in comparison to the Short‐read group (Figure S10, Supporting Information). This finding suggests that long‐ read possess a greater potential for recovering low‐abundance viruses than initially anticipated. Additionally, we detected significant disparities in length distributions among the three groups. Particularly, Long‐read and Both viruses were longer and exhibited a higher ratio of complete genomes than Short‐read viruses (Figure 1G). The genome length not only contributed to the assembly of more complete genomes (Figure 1H) but also played a crucial role in accurately assigning taxonomy to these vOTUs (Figure 1I). For instance, upon applying taxonomic classification to ≈78.52% of CHGV vOTUs using Demovir (https://github.com/feargalr/Demovir), VirusTaxo^[^ ^20^ ^]^ and PhageGCN,^[^ ^21^ ^]^ we observed that longer vOTUs were more easily matched to viral taxonomy (Figure S11, Supporting Information). Further investigate of the viral genomes’ host (based on the host information from the Virus‐Host DB) revealed that most of our recovered vOTUs are prokaryotic viruses (≈72%), while maintaining the ability to recover eukaryotic viruses (≈28%, Figure 1J).

Collectively, our findings underscore the significantly enhanced viral discovery potential of the combined sequencing approach compared to relying solely on short‐read methods.

### 2.3 Long‐Read Sequencing Helps Identify Multiple Virulent Phages That Are More Prevalent Than crassphages or Gubaphages

We next characterized the CHGV viruses by first examining their prevalence across our samples. It has been reported that the crAssphages and Gubaphages represent the most abundant and prevalent viral clades in the human gut.^[^ ^2^, ^24^ ^]^ A recent short‐read sequencing of VLP‐enriched samples further confirmed that crAssphage is the most prevalent phage at the strain level. As no virus was found to exceed the prevalence of the known most prevalent crAssphage in their research. In the CHGV, we also attempted to detect bacteriophages more prevalent than crAssphage at the strain level.^[^ ^2e^ ^]^ In the CHGV, we identified a total of 319 crAssphages and 223 Gubaphages (Table S2, Supporting Information). To establish the presence of a virus within a sample, we applied a comprehensive criterion: over 50% of the genome length should be covered by sequencing reads, with a sequencing depth exceeding 4X across the entire genome (Experimental Section). Our analysis revealed that, on average, crAssphages were present in 3.18% of the 135 samples, while Gubaphages were found in 8.60% of the samples. Notably, the most prevalent crAssphages were observed in 13% of samples, and similarly, the most prevalent Gubaphages were found in 20% of samples (Table S2, Supporting Information).

**Figure 2:**
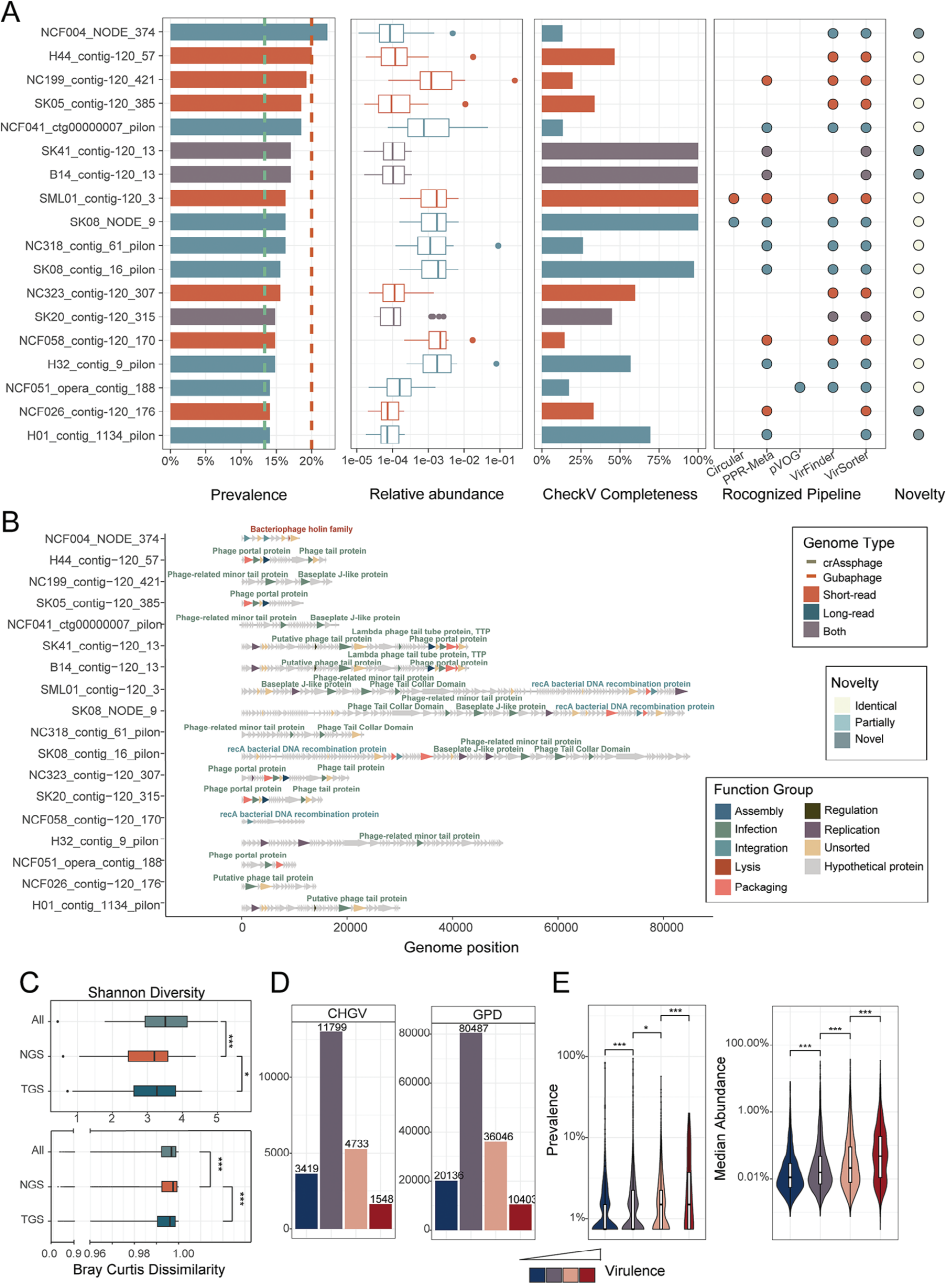
Characterization of the gut virome in CHGV facilitated by the combined assembly of short‐ and long‐read. A) Identification of gut phages that are more prevalent than the crAssphages or Gubaphages, and their characterization. Plots from left to right show the prevalence across 135 samples, relative abundance CheckV completeness, recognition by viral identification pipelines, and the novelty as compared with public viral databases; the bar colors indicate whether they could be obtained by Long‐read (dark blue), Short‐read (red) assemblies or both (grey). The vertical lines indicate the prevalence of the most prevalent Gubaphage (red) and crAssphage (green) identified in our samples, respectively. B) Genomes annotation of these highly prevalent phages. The line‐arrow charts show the genome annotation results of the corresponding viruses. The annotated protein‐coding genes (arrows) are colored according to their viral function, including lysis, infection and integration (Experimental Section). C) Within‐ and between‐ sample diversities of the CHGV viruses in our cohort. Upper panel, Shannon index. Bottom panel, pairwise Bray‒Curtis dissimilarities across the 135 samples with viral short‐read data. All: all CHGV virueses; Short‐read and Long‐read: CHGV viruses obtained by using short‐read only and long‐read (including long‐read and hybrid) assemblies. D) Lifestyle assignments of CHGV viral populations (VPs) according to the DeePhage tool; human gut vOTUs from the GPDwere used as a comparison. The vOTUs were classified into four categories according the DeePhage scores: temperate (with scores ≤0.3, dark blue), uncertain temperate (0.3–0.5, light blue), uncertain virulent (0.5–0.7, purple), and virulent (>0.7, red). Higher scores indicate higher virulence.^[^ ^33^ ^]^ E) Prevalence (left panel) and abundance (right panel) of CHGV viruses (at the VP level) as a function of virulence. Level of significance in C,D, and E: * *p* < 0.05, ** *p* < 0.01, ***: *p* < 0.001, Wilcoxon rank sum test.

Interestingly, we identified one virulent virus that exhibited higher prevalence than all Gubaphages, and an additional 17 virulent viruses were more prevalent than all crAssphages (Figure 2A). It's important to note that we excluded predicted temperate phages due to the potential overestimation of their prevalence caused by contaminating bacterial reads. All the identified crAssphages and Gubaphages in this study were classified as lytic phages (Table S2, Supporting Information), consistent with prior investigations.^[^ ^2^, ^24^ ^]^ Further functional annotation of these 18 super‐prevalent viruses for recognized viral proteins, such as those proteins associated with lysis, infection, or integration, provided additional validation of their viral identity (Figure 2B). Importantly, 61% of these highly prevalent vOTUs (11 out of 18) belonged to either the Long‐read or Both groups (Figure 2A), underscoring the substantial contribution of long‐read sequencing to their identification. Despite their lower abundance in comparison to the prevailing Gubaphage and crAssphage (Figure 2A; Figure S12, Supporting Information), these highly prevalent vOTUs affirmed our earlier observation that long‐read sequencing is adept at recovering low‐abundance gut viral genomes. Of note, 10 of the 18 super‐prevalent vOTUs were only partial according to CheckV (figure 2A). Additionally, 13 out of the 18 vOTUs could be found in other public datasets at the threshold of >70% ANI, with 4 being identical matches (i.e., >90% ANI; Table S3, Supporting Information). Interestingly, 4 out of the 10 fragmented vOTUs could found more complete homologous sequences in other databases (Table S4, Supporting Information), suggesting that the public databases could be used to further improve the quality of our dataset. We next attempted to annotate these vOTUs taxonomically and found that the majority of them could be assigned to the phylum Uroviricota (Table S5, Supporting Information). This phylum is structured into a single class (Caudoviricetes) and order (Caudovirales).^[^ ^25^ ^]^ For the four highly prevalent vOTUs that possess longer homologous sequences in other public datasets, especially the NC323_contig‐120_307. There has been a challenge in assigning it to a specific taxonomic category (Table S3, Supporting Information). Yet, the identification of a more complete homologous sequence in a public database has facilitated its classification within the Caudovirales class (Table S4, Supporting Information), confirming that longer contigs are more conducive to accurate taxonomy, consistent with the conclusions of our Figure 2I. We also employed both sequence similarity‐based methods and several host‐prediction tools including HoPhage^[^ ^26^ ^]^ and PHIAF^[^ ^27^ ^]^ to predict hosts for these super‐prevalent vOTUs and found that the confidence in the predicted results was generally low (Table S6, Supporting Information). The challenge of accurately predicting viral hosts hinders virome research, and more reliable future methods and strategies need to be developed in the future.

Subsequently, we conducted a more in‐depth analysis of the highly prevalent virus NCF004_NODE_374 (detected in ≈22% of the samples). This 11263 base pair (bp) viral genome encodes eleven proteins. Among these proteins, we identified two Peptidase family M23 proteins, two Transposase IS116/IS110/IS902 family proteins, one Bacteriophage holin family protein, and one N‐acetylmuramoyl‐L‐alanine amidase. All the four protein families are frequently found in bacteriophages according to the Pfam database,^[^ ^28^ ^]^ although the exact functions of the first family are yet to be experimentally validated. The Transposase IS116/IS110/IS902 family proteins are required for efficient transposition of the insertion sequence or transposon DNA and are essential in the process of integration of viral genomes.^[^ ^29^ ^]^ The Bacteriophage holin family proteins are needed for bacterial lysis and virus dissemination,^[^ ^30^ ^]^ while the N‐acetylmuramoyl‐L‐alanine amidases are responsible for catalyzing a chemical reaction that cleaves the linkage between N‐acetylmuramoyl residues and L‐amino acid residues in certain cell‐wall glycopeptides.^[^ ^30^, ^31^ ^]^ We further excluded the possibility that NCF004_NODE_374 was a prophage by comparing its nucleotide sequences against the UHGG genomes and the prophages in the Microbe‐versus‐phagedatabase^[^ ^32^ ^]^ (Experimental Section). These findings provide compelling evidence of its viral nature and lytic lifestyle. Furthermore, the bacterial hosts associated with this virus remain elusive, as neither the CRISPR‐spacer‐ nor the Trna‐based methods yielded any significant matches in the UHGG and other bacterial genome databases (Methods). Additionally, following the same criteria as illustrated in Figure 1E, it became apparent that NCF004_NODE_374 was distinct from the entries in public viral databases. In summary, these observations strongly suggest that NCF004_NODE_374 represents a novel viral clade that is prominently present within the human gut. Notably, the assembly of NCF004_NODE_374 was exclusively accomplished through the utilization of long‐reads (Figure 2A), providing further validation of the efficacy of our approach.

We next examined the diversity of the gut virome, both within individual samples and across all samples. As a metric of community complexity within samples, we employed the Shannon diversity index (also referred to as alpha diversity). Our analysis revealed significantly higher Shannon indexes across all CHGV viruses in contrast to the Short‐read group (Figure 2C). This observation signifies that the incorporation of long reads further enhanced the diversity of the gut virome within individual samples, primarily due to the identification of a greater number of vOTUs from each sample (Figure 1C). Conversely, when gauging dissimilarities between samples (quantified by Bray‐Curtis dissimilarity), we noted significantly lower values within all CHGV viruses in comparison to the Short‐read group (Figure 2C). This could be attributed to the diminished between‐sample dissimilarities observed in the Long‐read group. Given that the Long‐read and Both groups exhibited higher ratios of completeness in comparison to the Short‐read group (Figure 1H), our findings indicate that the previously documented widespread diversity and individual‐specific distribution of the gut virome^[^ ^9a^ ^]^ was, in part, a consequence of the fragmented nature of short‐read assembled viruses.

We also investigated the association between viral lifestyles and their distribution patterns among samples. Among the entire set of CHGV vOTUs, a substantial 70.78% were categorized as either temperate (3419) or held an uncertain temperate classification (11869). In contrast, a smaller 29.22% were classified as either uncertain virulent (4733) or unequivocally virulent (1548) (Figure 2D). Similar trends were observed in GPD and GVD datasets, which comprised 60% and 68% temperate or uncertain temperate phages, respectively (Figure 2D; Figure S13, Supporting Information). These findings highlight that the human gut virome primarily consists of temperate viruses, aligning with previous study outcomes.^[^ ^2^, ^6^ ^]^ Nonetheless, it's important to note that despite their lower numerical representation, virulent viruses were substantially more abundant overall. Specifically, the collective abundance of virulent viruses accounted for 58.66% of the total viral abundance, even though they made up only 29% of the total virus diversity (including both virulent and uncertain virulent viruses). Furthermore, we observed a direct relationship between virulence and both prevalence and mean abundances of the viruses (Figure 2E). In conclusion, our findings suggest that virulent viruses constitute the active and influential segment of the human gut virome. Their virulent lifestyle significantly contributes to their proliferation, both within individual samples and across all samples.

### 2.4 Long‐Read Sequencing Contributes to Most vOTUs of The Top Viral Clusters

Next, we examined the diversity of the CHGV vOTUs at higher taxonomic levels. Employing a procedure similar to the GPD database,^[^ ^2b^ ^]^ we organized the vOTUs into viral clusters (VCs)utilizing a graph‐based clustering algorithm known as Markov clustering (MCL)^[^ ^34^ ^]^ (Experimental Section). This process yielded a total of 1886 non‐singleton VCs. Among these clusters, 27 VCs consisted of at least 10 vOTUs, with the most extensive VC encompassing 31 viruses (Table S2, Supporting Information).

**Figure 3:**
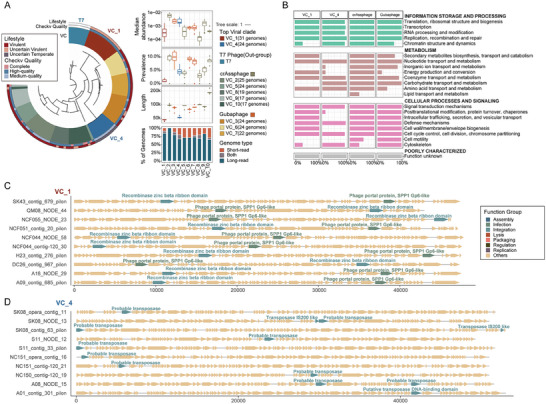
Taxonomic and functional characterization of the top VCs (CHGV) identified in CHGV. A) Phylogenetic analysis of the top ten VCs (ranked by VC size) using terminase protein sequences (left) and their abundance, prevalence, length and genome origin in our samples (right). The prevalence of the member phages was calculated using an arbitrary threshold to define the presence of a virus in a sample, i.e., the sequencing reads from the sample should cover >50% of the genome with >4X overall sequencing depth. B) The prevalence of protein function of each VC type. Protein function were classified according to the Pfam annotation and COG catalogue (Experimental Section). C,D) Genomes annotation of VC1 and VC4. The line‐arrow charts show the genome annotation results of virus in the corresponding viruses. The annotated protein‐coding genes (arrows) are colored according to their viral function, including lysis, infection and integration (Experimental Section).

The size of VCs often mirrors the diversity of corresponding clades,^[^ ^2b^ ^]^ representing the number of viral genomes (species or strains) present in a given environment. In line with this notion, we ranked the VCs based on their sizes and made an intriguing observation: among the top ten clusters, five were attributed to Gubaphages and three to crAssphages (Figure S14, Supporting Information). This aligns with earlier findings that established Gubaphages and crAssphages as the two most diverse and well‐known phage clades within the human gut.^[^ ^2b^ ^]^ Remarkably, we identified VC_1, which emerged as the most diverseVC in terms of containing the highest number of vOTUs. Importantly, this VC did not belong to either crAssphages or Gubaphages. Another notable discovery was VC_4, which surpassed the size of several crAssphage and Gubaphage clusters (Figure S14 and Table S2, Supporting Information). Both VC_1 and VC_4 formed their own distinct clades within a phylogenetic tree constructed using terminase genes (Experimental Section). The members of VC_1 and VC_4 showed high abundances that were comparable to that of crAssphages and Gubaphages, and were also prevalent, with a median prevalence of 0.7% and 4%, respectively. This is noteworthy, given that the median prevalence across all VCs in our samples stood at ≈0.7%. Both VC_1 and VC_4 possessed genome sizes of 40 kb and 50 kb, respectively (Figure 3A), and were classified as high‐quality (VC_1) or medium‐quality (VC_4) based on CheckV assessment (Figure 3A; Table S2, Supporting Information). Taxonomic annotation indicated that all vOTUs in VC_1 and most members in VC_4 belonged to various families under the order Caudoviricetes (Figure S15, Table S2, Supporting Information). Importantly, a significant portion of vOTUs within these VCs were attributed to the long‐read assembly (Figure 3A), emphasizing the substantial contribution of long‐read sequencing techniques in their identification.

We further explored the functional capacities of the protein‐coding genes of the top VCs. We annotated the identified viruses via hmmsearch^[^ ^35^ ^]^ v3.3.2 against the Pfam^[^ ^28^ ^]^ v34.0 database, and classified them into different function group according to the Clusters of Orthologous Groups (COGs; Methods). Particularly, vOTUs in VC_1 contained a greater number of bacteriophage‐related functional genes (Figure 3C; Figure S16, Supporting Information) compared to those in VC_4 (Figure 3D; Figure S17, Supporting Information). This is likely due to the higher genome completeness in VC_1 than the VC_4 (Figure 3A). Additionally, our analyses indicated that the crAssphages and Gubaphages contained more diverse gene functions, especially those related to in metabolism (Figure 3A, Table S7, Supporting Information).

However, the functional distribution was uneven among the vOTUs, likely influenced by the inclusion of several VCs within the top 10 clusters. In comparison to other VCs, crAssphages were more enriched in metabolic functions, particularly those less prevalent in other VCs, such as lipid transport and metabolism, nucleotide transport and metabolism, and cytoskeleton‐related functions (Figure S18, Supporting Information). Given that viruses typically don't possess their own metabolic genes, these findings suggest that crAssphages might employ these genes to complement host metabolic processes, ultimately benefiting their own survival. In contrast, viruses within VC_1 and VC_4 encoded fewer metabolic genes, potentially due to their smaller genome sizes (≈50 kb compared to ≈100 kb for Gubaphages and crAssphages). Intriguingly, genes associated with bacterial defense mechanisms were prevalent among the top 10 VCs, present in ≈approximately 50% of the vOTUs. Notably, DNA methylases and Type III restriction enzymes were the most frequently encountered, particularly within VC_1 (100% for restriction enzyme) and VC_4 (58% for DNA methylase) (refer to Table S7 for specifics). These two proteins typically form components of bacterial restriction‐modification (RM) systems. The restriction endonuclease recognizes a highly specific target DNA sequence (i.e., a distinctive, usually recurrent, molecular sequence, or motif) and degrades the unmethylated ones, while the corresponding DNA methyltransferase (Mtase) that protects the same DNA sequence via the DNA methylation of the bacterial genome.^[^ ^36^ ^]^ These results suggest that the viruses in the top VCs frequently hijacked the important component of the RM system^[^ ^37^ ^]^ to escape from host immune mechanisms for their own fitness benefits, which are consistent with our earlier observations that phage‐encoded DNA methylases significantly contributed to higher phage prevalence and abundances by protecting their genomes with DNA methylations.^[^ ^38^ ^]^

### 2.5 Quantifying Novelty in CHGV vOTUs at Higher Taxonomic Levels

**Figure 4:**
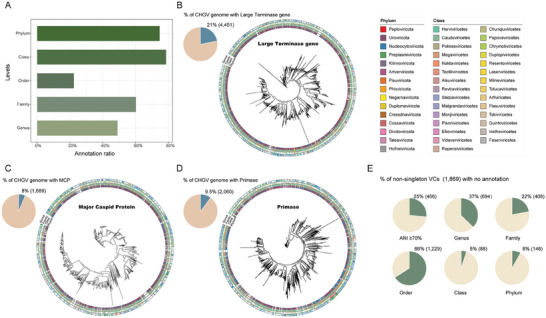
Quantifying Novelty in CHGV vOTUs at higher taxonomic levels. A) Bar plot showing the annotation ratio of the CHGV vOTUs using “marker‐based” methods at each taxonomic level. B,C,D) Phylogenetic relationships of CHGV vOTUs according to their encoded large terminase (B), major capsid protein (MCP, C) and primase (D) genes. The concentric color strips, progressing from the innermost circle to the outer circle, depict the “marker‐based” annotation results at the Phylum, Class, Family, and Genus levels. The pie chart situated in the upper left corner of each sub‐figure illustrates the proportion of the CHGV vOTUs containing the respective proteins. E) The pie charts depict the proportions of nsVCs lacking sequence similarity with public viral genomes or taxonomic annotations at the Genus, Family, Order, Class, and Phylum levels.

We next sought to quantify the novelty in the CHGV vOTUs at genus and higher taxonomic levels. Due to the lack of universally conserved gene markers, this task has been approached using either homologous sequences searching against taxon‐specific viral hallmark makers (“marker‐based” method) or whole‐genome‐based sequence clustering (“clustering‐based” method).^[^ ^2b^ ^]^ In this study, we first assigned 78.5% of the CHGV vOTUs into distinct taxonomic ranks (genus‐level and above) using three marker‐based methods, namely large terminase gene, major capsid protein and primase. At the genus level, 49% of the CHGV vOTUs could be assigned. The annotation rate generally increased with increasing taxonomic ranks, reaching to 74% at the phylum level. However, we were only able to assign 22% of the CHGV vOTUs to known Orders, likely due to the inherent incompleteness of the existing taxonomy lineage at NCBI (Figure 4A). Nevertheless, our results suggest that the CHGV contains substantial novelty at all taxonomic levels.

Individual marker genes including those coding for the large terminase gene, major capsid protein (MCP), and primase are also frequently found in viral genomes and used for phylogenetic analysis.^[^ ^12^, ^18^ ^]^ We thus also annotated these genes in the CHGV and used them to infer phylogenetic relationships of the corresponding vOTUs. Among the total, 4451 vOTUs (21%) were found to contain the large terminase gene, 1689 vOTUs (8%) contained MCP, and 2060 vOTUs (9.5%) contained primase (Figure 4B–D; Table S2, Supporting Information), the prevalence of which are consistent with the literature that “most single markers” are limited by low prevalence among viruses (<20%).^[^ ^39^ ^]^ Interestingly, the gene‐based phylogeny did not align well with the “marker‐based” taxonomic annotations, which was evident from the phylogenetic trees, where vOTUs in the same taxonomic ranks (i.e., Phylum, Class, Family, or Genus) often did not cluster together as anticipated (Figure 4B,C, and D). These results, along with the low prevalence of those marker genes in the overall virus population,^[^ ^39^ ^]^ highlight the challenges in identifying novel viral taxa in virology. However, there are many branches in the phylogenetic tree that cannot be annotated to corresponding taxonomic levels, and this lack is especially prevalent at the genus and family classification levels. These unassigned branches represent potential new families and genera.

Surprisingly, we observed significant agreements between the “clustering‐based” CHGV clusters and the marker‐gene based phylogeny, as evident from the terminase analysis of the top VCs(Figure 3A). We thus further explored quantifying the CHGV novelty at the genus (VC) level. We limited our analysis to a total of 1869 non‐singleton VCs (nsVCs). When compared to public (gut) viral databases,^[^ ^2^, ^6^, ^19^ ^]^ 25% of the nsVCs lacked sequence similarity (i.e., <70% ANI) to their contained vOTUs. When compared to the “marker‐based” annotation results, 37% of the nsVCs did not have any genus‐level annotations, while 8% were not annotated at the Phylum level (Figure 4E). These results indicate that CHGV could potentially contain at least hundreds of novel genera and a few phyla, although the annotation at higher taxonomic levels beyond genus should be further validated using computational and experimental approaches.

### 2.6 Functional Annotation of All and Novel CHGV vOTUs

We then proceeded to functionally characterize all GHGV vOTUs. We quantified the number of proteins allocated to each of the 23 COGscategories (Table S7, Supporting Information). Our focus was on delineating the functional capabilities of novel vOTUs (i.e., the 6962 viruses exhibiting less than 70% genomes similarity with public catalogues). Through this analysis, we identified a total of eight enriched categories (as determined by Chi‐Squared Test, p<0.001; Experimental Section), in comparison to those present in publicly available virome databases (Figure 1E, Experimental Section).

**Figure 5:**
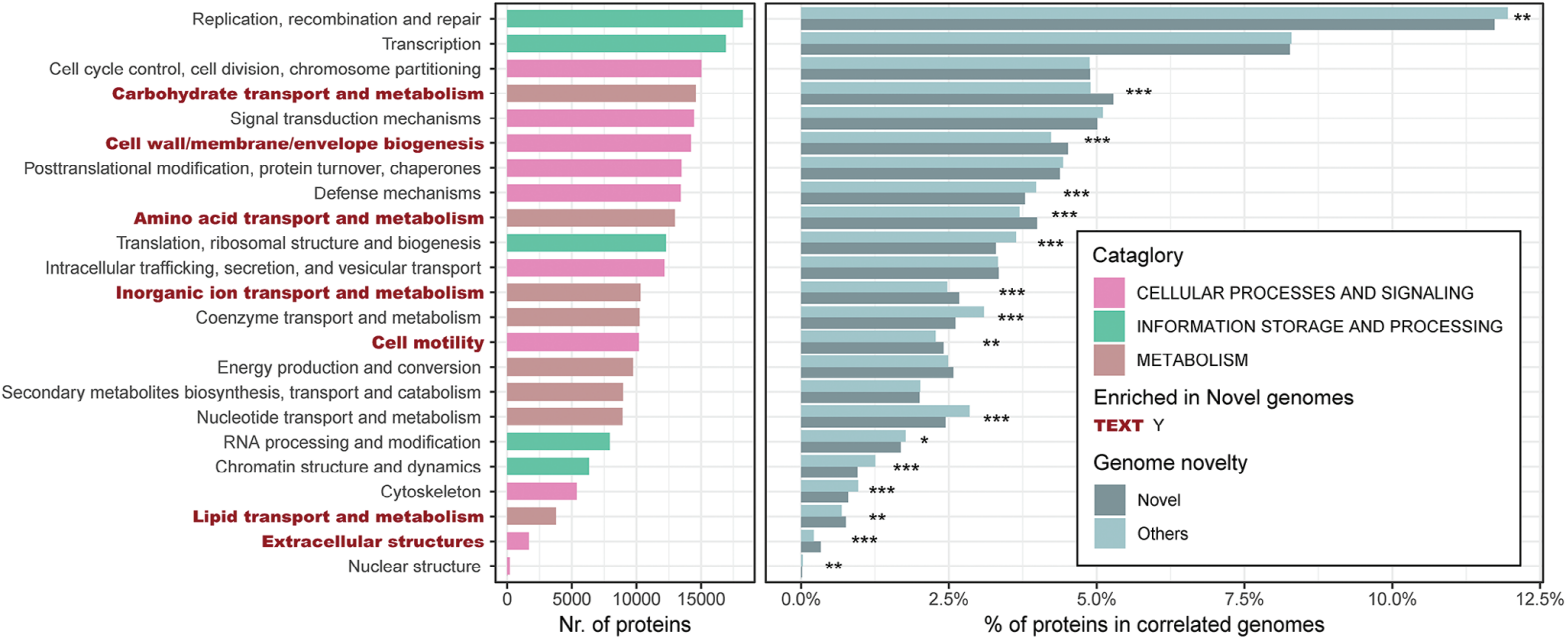
Functional characterization of the novel vOTUs as compared with the others in the CHGV catalog. Left: number of proteins in all CHGV vOTUs assigned to each of the 23 COG functional categories; the red texts indicate those that were enriched in the novel vOTUs as compared with the others in the CHGV catalog, while the colors of the bars indicate the corresponding broad COG categories. Right: proportions of the annotated proteins of each functional category in the novel and other vOTUs. Level of significance: * *p* < 0.05, ** *p* < 0.01, *** *p* < 0.001; Chi‐Squared Test.

An intriguing observation emerged that among these enriched categories, five belonged to the “METABOLISM” group. These categories encompassed Carbohydrate transport and metabolism, Amino acid transport and metabolism, Inorganic ion transport and metabolism, Energy production and conversion, and Lipid transport and metabolism (Figure 5, highlighted in dark red text). Additionally, three enriched categories pertained to CELLULAR PROCESSES AND SIGNALING, encompassing Cell wall/membrane/envelope biogenesis, Cell motility, and Extracellular structures (Figure S19, Supporting Information).

Given that metabolic functions are often considered non‐essential for viruses, our findings hint at a potential propensity among novel gut viruses to be involved in the metabolic processes of their bacterial hosts. However, further experimentation is imperative to validate the roles of viral‐encoded metabolic genes in both viral and host fitness.

### 2.7 Bacterial Host Assignments for CHGV Viruses and Construction of A Virus‐Bacteria Network

Viruses have rather narrow host ranges^[^ ^32^, ^40^ ^]^ and thus are an ideal tool for precision manipulation of gut microbiota. We thus assigned bacterial hosts to the VPs through a customized bioinformatics pipeline by utilizing CRISPR (clustered regularly interspaced short palindromic repeats)‐spacer‐bacteria and tRNA‐bacteria relationships by searching for homologous sequences between the CHGV vOTUs and the CRISPR‐spacers and/or tRNAs in the bacterial genomes (Experimental Section); both methods have been used to establish virus‐bacteria relationships.^[^ ^41^ ^]^

**Figure 6:**
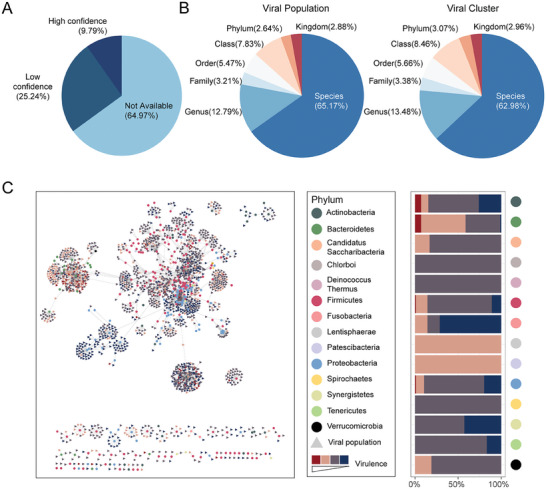
Bacterial host assignments for CHGV viruses. A) Host assignment confidence distributions. Here high‐, and low‐confidence represents virus‐host relationships supported by multiple evidence (i.e., > = 2 spacers, > = 2 tRNAs, or > = 1 spacers and > = 1 tRNAs), and others were considered as of low confidence. B) Host range distributions of the CHGV viruses at the VP‐ (left panel) and VC‐ (right panel) levels. The host range of a virus was calculated as the last common ancestor of all its predicted hosts on the NCBI taxonomic tree. C) Phage‐host interaction network (left panel) between VCs and bacterial hosts at the genus level visualized using Cytoscape.^[^ ^43^ ^]^ Dots represent bacterial genera and are colored by their corresponding phyla. Solid triangles represent VCs and are colored according to their virulence. Virulence was predicted using the DeePhage tool (Experimental Section). Lifestyle preference (right panel) of the viruses associated with the bacterial phyla. Y‐axis: bacterial hosts at the phylum level; X‐axis: lifestyle compositions of viruses associated with the bacterial phyla.

In total, we assigned 2866 bacterial species as hosts to 7583 (35.03% of the total 21499) vOTUs that had at least one matching spacer or tRNA sequence (Table S5, Supporting Information). Among these, the host assignments for 2119 vOTUs were supported by more than one spacer and/or tRNA matches, corresponding to 1241 bacterial species in total; we considered these as high‐confidence interactions and only included them into the subsequent analysis (Figure 6A; Experimental Section; Table S8, Supporting Information). Most of the vOTUs had narrow host ranges at the species (65.17%) and genus (12.79%) levels (Figure 6B), consistent with previous findings.^[^ ^32^, ^40^ ^]^ We found similar results at the VC levels (≈62.98% and ≈13.48%), suggesting members of the same VC likely had the same hosts. Including interactions of lower confidence led to broader host ranges (Figure S20A; Table S5, Supporting Information). It is important to acknowledge that CRISPR spacers exhibit high dynamism and may be lost over time. Our analysis did not encompass the assembly of bacterial genomes from the same samples for CRISPR identification, potentially accounting for the absence of spacer matches due to this dynamic nature. However, this does not necessarily imply the complete absence of a CRISPR system within the bacterial host. In terms of taxonomic distribution, the bacterial phylum Firmicutes encompassed 54.10% of the total assignments. At the bacterial genus level, Clostridium exhibited interactions with the highest number of VCs per genus (Figure S20B, Supporting Information), followed by Bifidobacterium, Bacteroides, and Ruminococcus. Notably, Bacteroidetes displayed a higher viral diversity, averaging 17.7 VCs per genus. In general, more prevalent bacterial clades showed associations with a greater number of vOTUs and VCs (Figure S20C).

We also investigated whether the bacterial hosts had preferred virus lifestyles (Figure 6C). We found that at the genus level, most genera preferred temperate virus; among the 194 genera with at least two viruses, only 2 have a virulent‐to‐temperate (V/T) ratio greater than one. For example, the viruses associated with *Prevotella* had the highest V/T ratio of 2.59, followed by *Bacteroides* (1.70). At the phylum level, *Bacteroidetes* associated viruses had the highest V/T ratio (1.41). Since we are at a very early stage of gut virus discovery and rely mostly on predictions to establish phage‐host interactions,^[^ ^42^ ^]^ these results and our interpretations should be further validated with more data.

## 3 Discussion

The effective and unbiased discovery of viral genomes plays a pivotal role in unraveling and comprehending the intricacies of the human gut virome.^[^ ^44^ ^]^ In this study, we present a pioneering large‐scale exploration of the human gut virome through the integration of both long‐ and short‐read sequencing approaches. This endeavor yielded a collection of 21499 high‐quality, non‐redundant human gut viruses. Notably, our method exhibited heightened efficiency (≈35%) in acquiring complete viral genomes compared to existing public databases. This enhancement holds the potential to significantly expedite our efforts in functionally exploring these phages. Remarkably, a substantial majority (∼60%) of the CHGV vOTUs were successfully obtained through long‐read or hybrid (utilizing both long‐ and short‐reads) assemblies, rather than relying solely on short‐read assembly. This observation underscores the greater contribution of long‐read sequencing in capturing gut viral genomes. The reasons behind the inability to assemble the majority of genomes solely through short‐reads remain unclear. We hypothesize that shorter contig lengths resulting from short‐read assembly might be a contributing factor, as longer contigs stand a better chance of being recognized as viral entities (Figure S1, Supporting Information). Additionally, viruses are known to exhibit higher microdiversities (indicating genetic variation within the same viral species) in comparison to bacteria,^[^ ^9^, ^45^ ^]^ rendering them challenging to assemble using short‐reads.^[^ ^46^ ^]^ Our findings highlight the potential of long‐read sequencing in overcoming these challenges and advocate for the essential role of combined sequencing approaches in unearthing the gut virome. Our results are consistent with recent publications that long‐read sequencing can help generate complete bacterial genomes from human (fecal) metagenomes.^[^ ^47^ ^]^ Of course, the choice of assembly software used during the assembly process can also affect the results.^[^ ^48^ ^]^ The advantages obtained through our methodology inherently result from the co‐influence of the sequencing technology and the assembler. However, in practical applications, it is challenging to completely disentangle the two factors for a separate discussion. Especially, when it comes to long‐read sequencing assembly and hybrid assembly, where the assembly program is always closely integrated with the sequencing technology employed.

Digging deeper into the CHGV vOTUs, we unveiled concealed layers of diversity within the human gut virome, predominantly brought to light by the extended capabilities of long‐read sequencing. In various aspects, our exploration yielded notable revelations. For instance, we unearthed several virulent bacteriophages that exhibited higher prevalence than even the most dominant crAssphage and/or Gubaphage (Figure 2A), two phage categories that have thus far been recognized as the most diversified within the human gut.^[^ ^2^, ^24^ ^]^ Additionally, we identified two VCs showcasing greater diversity than the crAssphages and/or Gubaphage. Most intriguingly, a substantial proportion of these vOTUs were effectively recovered through the application of long‐read sequencing (whether in the form of long‐read assemblies or through hybrid methods), further reinforcing the significance of combining these sequencing strategies for a comprehensive gut virome analysis. The outcomes of our study also offer compelling insights: they indicate that the human gut virome likely exhibits even greater diversity than previously envisioned. This underscores the importance of ongoing research and underscores the potential of long‐read sequencing to uncover new dimensions of complexity within the gut virome landscape.

Our findings hold two significant implications. First, despite the remarkable strides made in recent times toward comprehending the human gut virome on a larger scale,^[^ ^2^, ^6^ ^]^ we are just scratching the surface in terms of uncovering its true diversity. This ongoing quest is accompanied by notable challenges, both theoretical and technological in nature. Theoretical hurdles include the absence of universally applicable viral marker genes, which renders the identification and quantification of novel viral genomes a complex endeavor.^[^ ^49^ ^]^ It is widely accepted that quantifying the novelty of uncultured viral genomes relies heavily on sequence similarity and phylogenetic inference.^[^ ^50^ ^]^ Sequence similarity is commonly detected using pairwise sequence alignment and generally yield classifications largely congruent with those of the International Committee on Taxonomy of Viruses.^[^ ^39^, ^51^ ^]^ However, phylogenies constructed from single marker genes, such as the terminase, major capsid protein, and primase genes, often have limited phylogenetic signals due to the low prevalence of these genes.^[^ ^39^ ^]^ While these methods provide valuable insights into relationships at the species and genus levels, there remains a lack of quantifiable approaches for higher‐level viral taxonomy classification. Hence, developing computational tools for viral taxonomy is vital to advance this research field.^[^ ^52^ ^]^ On the technological front, the formidable presence of abundant proteins and polysaccharides in human feces poses constraints on viral DNA yields.^[^ ^53^ ^]^ Moreover, short‐read sequencing methods often falter in capturing the full‐length genomes of sizable viruses. In this study, we tackled some of these challenges by leveraging long‐read sequencing techniques on viral‐like particle‐enriched fecal samples. However, the acquisition of sufficient viral DNA necessitated the collection of ≈500 g of feces from each participant, a task that is logistically and practically demanding. Hence, there exists a pressing need for the development of efficient VLP extraction methodologies tailored to human feces, coupled with long‐read sequencing approaches that demand lower quantities of viral DNA. However, it's important to acknowledge a limitation of our study arising from the substantial amount of starting material used. While this approach provides valuable insights into the human gut virome, it may pose challenges when applying the method to investigate other ecosystems, such as mice or infant fecal microbiota. The requirement for a large volume of starting material may limit the feasibility and practicality of extending our method to these distinct ecosystems. Further innovations in sample collection, processing, and sequencing could potentially address this limitation and broaden the scope of virome analysis across diverse environments. Addressing these challenges is pivotal in facilitating a more comprehensive exploration of virome, ensuring that the full extent of its diversity is fully unveiled and harnessed for further scientific insights.

Second, it is evident that novel theoretical and analytical frameworks are essential to characterize virome ecology in ways distinct from those applied to bacteria. This necessity stems from the fundamental disparities between viruses and bacteria in terms of key characteristics. One such critical distinction arises in the calculation of prevalence, a metric frequently computed by considering all genomes identified within a particular environment. However, for bacteriophages, a viral genome typically exists in two distinct forms within the human gut: the lysogenic state (prophage) and the free‐particle state (VLP). Prevalence calculations for the prophage form tend to yield overestimations due to its integration with bacteria, rendering it inherently dissimilar from the free‐particle state. This discrepancy indicates the importance of adopting a nuanced approach to account for these diverse viral states during prevalence analysis. Moreover, conventional benchmarks such as a relative abundance of 1e‐4 (or 0.01%) often used to ascertain the presence/absence of a bacterium might not be readily applicable to viruses. This benchmark is predicated on the notion that a specific quantity of bacterial cells is requisite for functional significance. However, viruses span a far broader spectrum of taxonomic groups and can effectively function at considerably lower abundances, such as a Multiplicity of Infection (MOI) of 1e‐6. Although potential solutions such as categorizing bacteriophages into distinct lifestyles and the application of ultra‐deep sequencing have been proposed,^[^ ^12b^ ^]^ it remains evident that novel theoretical and analytical frameworks are indispensable to comprehensively characterize the intricate landscape of gut virome ecology. This endeavor is crucial in untangling the complex interplay between viruses and their bacterial hosts within the human gut ecosystem.

## 4 Experimental Section

### Sample Collection

Human fecal samples were obtained from healthy volunteers recruited in Wuhan and Shanghai, China. All volunteers remained anonymous but were asked to complete a questionnaire to collect relevant information such as their sex, age, height, weight, health status, and recent antibiotic usage (Table S1, Supporting Information). The exclusion criteria included (1) the use of antibiotics or probiotic supplements up to one month before the study; (2) the use of drugs known to significantly affect the gut microbiota composition, such as metformin,^[^ ^54^ ^]^ statin^[^ ^55^ ^]^ or proton‐pump inhibitors,^[^ ^56^ ^]^ in the month prior to sample collection; (3) current chronic intestinal diseases or a history of intestinal diseases; and (4) menstruation at the time of sampling in females. After collection, the samples were immediately cooled with dry ice and transferred to a −80 °C freezer within five hours. To obtain a large amount of feces for phage extraction, up to three stool samples were collected from each participant and mixed; the mixed samples totaling at least 500 grams were processed further. In total, 135 qualified samples were obtained (Table S1, Supporting Information).

This study was approved by the Ethics Committee of the Tongji Medical College of Huazhong University of Science and Technology, Wuhan China (No, S1241) and the Human Ethics Committee of the School of Life Sciences of Fudan University, Shanghai China (No, BE1940).

### Virome Enrichment and Short‐ and Long‐Read Sequencing

The virome enrichment protocol applied to the fecal samples was adapted from ref. [11c] with modifications to accommodate the large quantity of the collected feces from each participant. Briefly, 400–500 g of frozen feces taken from a −80 °C freezer was added to five liters of SM (200 mM NaCl, 10 mM MgSO4, 50 mM Tris‐HCl (pH 7.5)) buffer and stirred by an automated stirrer (A200plus, OuHor, Shanghai, China) at low speed (120 rpm) at room temperature until all feces were dispersed. Then, the suspended mixture was filtered through four layers of gauze (21 s x 32 s/28×28) and centrifuged at 5000 x g for 45 min at 4 °C. The supernatant was transferred to fresh tubes and centrifuged at 8000 x g for 45 min at 4 °C. The supernatant was subsequently concentrated to ≈300 ml via a 100 KD ultrafiltration membrane (Sartorius, VIVO FLOW 200). NaCl was then added to the filtrates to a final concentration of 0.5 mol L^−1^, and the samples were stored at 4°C for one hour. Then, PEG 8000 was added to a final concentration of 10% w v^−1^, and the samples were incubated at 4°C overnight. On the following day, phage particles were sedimented at 13 000 x g for 35 min at 4 °C.

The obtained pellets were fully suspended in 18–36 mL TE buffer and treated by gently shaking with an equal volume of chloroform. The mixture was centrifuged at 3500 x g for 10 min at 4°C. The aqueous phase was then transferred to a sterile round‐bottomed flask and evaporated for 15 min using a rotary evaporator at room temperature to remove traces of chloroform, which could affect the activity of DNase I in the subsequent step. The aqueous phase was transferred to a new centrifuge tube, TE buffer was added to recover the volume before treatment with chloroform, and DNase buffer was added to a 1× final concentration. Then, for every 6 mL of supernatant, 50 µL of a DNase I mixture (33.3 U µL^−1^, Biolab) and 25 µL of an RNase A mixture (0.5 U µL^−1^, Biolab) were added, and the resultant mixture was incubated in a thermostatic oscillator (THZ‐C, Peiying, Suzhou, China) at 100 rpm for 30 min at 37 °C before the enzymes were inactivated by the addition of EDTA buffer (final concentration 35 mM) and incubation at 70 °C for 10 min.

Nucleic acid was then extracted using a HiPure HP DNA Maxi Kit (D6322, Magen, Guangzhou, China) according to the manufacturer's instructions. Briefly, proteinase K and SDS lysis buffer were added, and the mixture was then incubated at 56°C for one hour. Viral particles were further lysed by adding the CFL buffer provided with the kit, and the lysates were subsequently treated with an equal volume of phenol:chloroform:isoamyl alcohol (25:24:1, pH 8.0), followed by centrifugation at 12 000 × g for 15 min at room temperature. After centrifugation, the supernatant was transferred to a new centrifuge tube and treated with an equal volume of chloroform with gentle shaking, followed by centrifugation at 12 000 x g for 15 min at room temperature. The aqueous phase was transferred to a new tube, loaded onto a DNA Mini Column provided by the kit, and centrifuged at 12 000 x g for 1 min. The DNA Mini Column was then washed with GDP and GW2 buffers. DNA was eluted using DNA elution buffer and stored at −80°C for further analysis. Note that all buffers and columns used in this part of the study were provided in the kit.

The purified VLP DNAs were quality checked and subsequently sequenced on the Illumina (short‐read) and PacBio (long‐read) platforms. For Illumina sequencing, nucleic acids were sheared with a g‐TUBE (Covaris, USA) to generate a target size fragment of 400 bp, followed by sequencing library construction using the Nextera XT DNA Library Preparation Kit (Cat. No. FC‐131‐1096, Illumina, USA) according to the manufacturer's instructions and sequencing using an Illumina HiSeq2000 sequencer (Novogen, Beijing, China) to generate paired‐end reads of 150 bp. The generated dataset was then referred to as viral short‐read sequencing data. For PacBio sequencing, DNAs were sheared into ≈5 kb fragments by using a g‐TUBE (Covaris, USA) and purified with AMPure PB magnetic beads, followed by a quality check using 0.7% agarose gel electrophoresis. The qualified samples were employed to construct sequencing libraries using the SMRTbellTM Express Template Prep Kit 2.0 (Pacific Biosciences, USA) according to the manufacturer's instructions. The quality of the DNA libraries was checked with an Agilent 2100 Bioanalyzer (Agilent Technologies, USA), and the libraries were then sequenced with a PacBio RS II sequencer (Pacific Biosciences, Menlo Park, CA, USA) in circular consensus sequencing (CCS) mode. The generated dataset was then referred to as long‐read sequencing data.

### Raw Data Processing

Raw Illumina short‐read of viral reads (referred to as viral short‐read hereafter) were processed with Trimmomatic v0.38^[^ ^57^ ^]^ (with parameter LEADING:3 TRAILING:3 SLIDINGWINDOW:15:30 MINLEN:50) to remove adaptors and trim low‐quality bases; reads of 50 bp or less after trimming were discarded. The PacBio long‐read sequencing of viral reads (referred to as viral long‐read hereafter) were corrected with CCS using pbccs (v4.0.0, https://github.com/nlhepler/pbccs) with the default parameters.

Putative human reads were identified from the trimmed/CCSed reads by aligning the latter to the human reference genome (hg38; GCA_0 00001405.15) using Bowtie2^[^ ^58^ ^]^ (v2.4.2, –end‐to‐end) with default parameters and removed from further analysis.

In total, 4.89 terabytes of clean data were obtained for the viral short‐read samples and 561 gigabytes of CCSed data for the viral long‐read samples.

### Removal of Bacterial Reads from Virome Sequencing Datasets

To evaluate bacterial contaminations, Bowtie2^[^ ^58^ ^]^ (v2.4.2) was used with default parameters to map the clean reads from the viral short‐read and viral long‐read datasets to the UHGG^[^ ^59^ ^]^ (Unified Human Gastrointestinal Genome) genomes. To prevent over‐estimation of the contamination, possible prophage regions were identified using PhageFinder^[^ ^60^ ^]^ (v2.1) and removed from UHGG genomes. The resulting UHGG dataset was referred to as UHGG‐Minus in this study. The contamination rate was then calculated for each sample as the percentage of reads (read pairs for the viral short‐read, and CCS reads for the viral long‐read data) aligned to the UHGG‐Minus genomes. The mapped reads were removed from further analyses to remove putative bacterial contaminations.

### Combined Assembly of Short‐ and Long‐ Read

Briefly, IDBA‐UD^[^ ^61^ ^]^ (Release 1.1.3, parameters: –maxk 120 –step 10 –min_contig 1000) was used to assemble the filtered viral short‐read data. Canu^[^ ^62^ ^]^ (v2.0‐, parameters: genomeSize = 20k corOutCoverage = 1 ‐corrected) and Flye^[^ ^63^ ^]^ (v2.8.2, parameters: –meta –genome‐size 20k –min‐overlap 1000) were used to assemble the filtered viral long‐read CCS reads. Because Canu does not have a meta‐assembly mode and tends to extend contigs by merging DNA sequences from different viral species to generate erroneous contigs, unitigs were used for subsequent analysis; unitigs were basic blocks of contigs that were shorter but more reliable than contigs (“unitigs” were derived from contigs; wherever a contig end intersects the middle of another contig, the contig was split).^[^ ^64^ ^]^ To further extend the sequences, MetaBAT2^[^ ^65^ ^]^ (version 2, default parameters) was used to group unitigs into bins. If all unitigs from one contig could be grouped into the same bin, contigs instead of unitigs were used for further analysis. OPERA‐MS^[^ ^66^ ^]^ (v0.9.0, parameters: ‐contig‐len‐thr 1000 –polishing –no‐strain‐clustering –no‐ref‐clustering) and metaSpades^[^ ^67^ ^]^ (v3.13.1, default parameters) were employed for hybrid assemblies using both the viral long‐read and viral short‐read datasets from the same samples (Figure S13, Supporting Information).

All contigs were analyzed by metaMIC^[^ ^68^ ^]^ for misassembly identification and correction; misassembled contigs were split by the tool.

Contigs/unitigs obtained from all the above three strategies were merged; for samples that did not have vial long‐read data, contigs from the IDBA‐UD assembler were used.

The merged dataset was dereplicated using CD‐HIT^[^ ^69^ ^]^ (v4.8.1, parameters: ‐c 0.95 ‐n 8) using a global identity threshold of 95%.

### Assessment of COBRA and VAMB for Viral Improvement and Binning

The assessment was initiate by subjecting all 135 short‐read samples to COBRA, a software designed for enhancing NGS viral assemblies (COBRA^[^ ^70^ ^]^ was only tested on NGS data as per its preprint). COBRA yielded improvements for just two out of the 21499 viral contigs utilized as input in the study.

Subsequently, we evaluated the performance of the VAMB^[^ ^71^ ^]^ tool by utilizing it for binning the contigs. Leveraging VAMB's capability for cross‐sample binning, data from all the Short‐read samples were input into the software, resulting in a total of 9432 bins (Table S9, Supporting Information). Among these, 4347 bins (42.5%) contained two or more contigs. To assess binning quality, the agreement was scrutinized of taxonomic annotations among multiple contigs within a bin. Strikingly, out of these multi‐contig bins, 3590 were annotated by the three aforementioned tools. However, merely 1220 bins (34%) exhibited contigs belonging entirely to the same family, while 2370 bins (66%) contained contigs from different families and higher taxonomic levels (Figure S21, Supporting Information).

While recognizing the utility of both COBRA and VAMB, it was apparent that their application did not align seamlessly with the distinct characteristics of the data. It was speculate that the modest improvement by COBRA may stem from its lack of testing on viral‐metagenome samples (as indicated in the COBRA preprint paper, https://www.biorxiv.org/content/10.1101/2023.05.30.542503v2). Additionally, VAMB was initially designed for bacteriome analysis (https://doi.org/10.1038/s41587‐020‐00777‐4) and was not optimized for binning the gut virome.

### Prediction of Viral Contigs With State‐Of‐The‐Art Tools and Removal of Potential Bacterial Contigs

To identify viral contigs, six independent state‐of‐the‐art viral identification pipelines were used. These were: (1) VirSorter v2.0^[^ ^72^ ^]^ (–min‐score 0.7), (2) VirFinder v1.1^[^ ^5b^ ^]^ (default parameters), (3) PPR‐Meta v1.1ART (default parameters).Furthermore, (4) A nucleotide‐level BLAST search was also conducted against the Viral RefSeq genomes using BLASTn v.2.7.1^[^ ^73^ ^]^ with the default parameters and an *E*‐value cutoff of <1e‐10; Release 201 (Jul 06, 2020) of the Viral RefSeq database contained 13148 viral genomes. (5) For protein‐level similarity searches, the annotated protein sequences were used for BLAST searches against the NCBI POG (Phage Orthologous Groups) database 2013.^[^ ^74^ ^]^ (6) CheckV was employed to determine the completeness of the virus.

A contig was annotated as a virus if it was circular/met at least two out of the following criteria 1–5, adopted from theGVD^[^ ^6^ ^]^:

1. VirSorter score ≥ 0.7,
2. VirFinder score > 0.6,
3. PPR‐Meta phage score > 0.7,
4. Hits to Viral RefSeq with > 50% identity & > 90% coverage,
5. Minimum of three ORFs, producing BLAST hits to the NCBI POG database 2013 with an E‐value of ≤ 1e‐5, with at least two per 10 kb of contig length.
6. Alternatively, contigs met one of the above criterium and were annotated as high‐quality (≥ 90% completeness) by CheckV^[^ ^22^ ^]^ were also annotated as viruses.

For some sequences might be shared between bacteria and bacteriophages, the removal of bacterial reads might not be enough. a BLAST search was thus carried out against the UHGG‐Minus sequences using BLASTn v.2.7.1^[^ ^73^ ^]^ with the default parameters and an *E*‐value cutoff of <1e‐10, and contigs with blastn hit of 90% identity over 50% of its length were removed from further analysis.

As short contigs may only represent fragments of viral genomes, contigs that were longer than 5 kb were selected for further analyses; this dataset was referred to as the CHGV dataset, which consisted of a total of 21499 viral populations.

Rarefaction curves were generated by randomly resampling the pool of N samples 10 times and then plotting the number of dereplicated (unique) contigs found in each set of samples.

### Taxonomy Assignment of CHGV vOTUs

To taxonomically classify the vOTUs, three distinct annotation tools were employed. VirusTaxo (https://github.com/omics‐lab/VirusTaxo, downloaded on 19th April 2022)^[^ ^20^ ^]^ used to compare the nucleotide sequences against its prebuilt database of VirusTaxo and assign them to a known viral genus at an entropy index threshold of <0.5. For those viruses not annotated by VirusTaxo, Demovir(https://github.com/feargalr/Demovir) was employed to search and compare predicted protein sequences with the TrEMBL virus protein database. Finally, for any remaining unannotated viruses, the PhageGCN was used,^[^ ^21^ ^]^ a model based on convolutional neural networks, to perform taxonomic assignment using default parameters. The three software outputs were integrated as the annotation results for CHGV.

### Public Viral Genome Databases/Catalogs Used in This Study

The following public human virome databases were used in this study. GPD^[^ ^2b^ ^]^ contains 142000 vOTUs assembled from metagenome sequencing. GVD^[^ ^2a^ ^]^ contains 33242 vOTUs assembled from Viral like particles (VLP) sequencing. MGV^[^ ^2c^ ^]^ contains 54118 candidate viral species assembled from metagenome sequencing. CHVD^[^ ^2d^ ^]^ contains 45033 viral taxa assembled from metagenome sequencing. DEVoC^[^ ^2e^ ^]^ contains 12986 vOTUs assembled from VLP sequencing. The NCBI viral Reference genomes, Release 201 (Jul 06, 2020) of the Viral RefSeq database contains 13148 viral genomes.

### Identification of Complete Phage Genomes in CHGV and Public Viral Datasets

The CheckV^[^ ^22^ ^]^ tool were used on the CHGV and public viral datasets, those that were annotated with 100% completeness were considered to be complete genomes (CheckV complete).

In addition, a customized pipeline was used to identify circular contigs that were considered as complete genomes in CHGV. First, the BLASTn program^[^ ^73^ ^]^ was used to search for alignable regions within each contig; if the front and tail portions of the contig were exact matches over 30 base pairs (nucleotide identity = 100, *E*‐value<1e‐5), they were considered as circular genomes.^[^ ^18^ ^]^ Second, the clean sequencing reads were mapped to the CHGV vOTUs using either pbmm2 (https://github.com/PacificBiosciences/pbmm2) for the viral long‐read data or bowtie2^[^ ^58^ ^]^ for the viral short‐read data. Genomes with at least two reads mapped to both the front and tail of the genome with over 50 bp hit length were considered to be circular genomes, resulting additional 1295 circular genomes.

### Estimating the Proportion Of Novel vOTUs In One Dataset As Compared With All Other Viral Databases

To estimate the proportion of novel vOTUs in one dataset, the BLASTn tool was used to search all its sequences against all other viral databases mentioned above. ANI was calculated by merging the hit regions with identity ≥95%, and hit length ≥ 500 bp, then calculated the coverage of these regions. Based on the overall ANI, a viral sequence was identical, partial identical or novel if it has ≥ 95%, ≥ 70% or <70% ANI as compared with other viral sequences.

### Functional Annotation of CHGV proteins

The encoded protein sequences of the CHGV vOTUs were annotated using Prodigal^[^ ^75^ ^]^ v2.6.3 with default parameters.

Proteins translated from the CDS sequences were then annotated with eggNOG mapper v1.0.3‐3^[^ ^76^ ^]^ and hmmscan^[^ ^35^ ^]^ v3.3.2 against Pfam^[^ ^28^ ^]^ v34.0, and VOGdb v204 (*E* value <1e‐5, score > = 50, http://vogdb.org/).

The terminase protein sequences were extracted to conduct phylogenetic analysis (below section).

Twenty‐five small function groups were classified based on the classification of functions in COGs,^[^ ^77^ ^]^ and then categorized into four categories, including INFORMATION STORAGE AND PROCESSING, CELLULAR PROCESSES AND SIGNALING, METABOLISM and POORLY CHARACTERIZED. *Note that each protein might be associate with multiple function groups or categories.

Eleven classes of phage parts were categorized based on their functions, including LYS (lysis), INT (integration), REP (replication), REG (regulation), PAC (packaging), ASB (assembly), INF (infection), EVA (immune evasion), HYP (hypothetical protein), UNS (unsorted), and tRNA according to a previous study.^[^ ^78^ ^]^

### Phylogenetic Analysis of Selected Phages

Phylogenetic analysis was performed for selected phages using the terminase protein sequences. Briefly, for each group of phages of interest, their terminase protein sequences were aligned using MUSCLE^[^ ^79^ ^]^ v3.8.1551 with the default parameters. Phylogenetic trees were built with FastTree^[^ ^80^ ^]^ v2.1.10 with default parameters. Phylogenetic trees were then visualized and annotated using iTol^[^ ^81^ ^]^ and EvolView.^[^ ^82^ ^]^

### Clustering Viral Contigs Into VCs

The clustering of gut viral contigs into VCswas performed using a strategy adopted from the GPD.^[^ ^2b^ ^]^ Briefly, a BLASTn algorithm with default parameters was used to search the nucleotide sequences of the CHGV viral contigs against themselves for homologous sequences. An *E*‐value threshold of 1E‐10 was first used to filter the BLASTn results; the BLASTn query‐hit pairs were further filtered to retain those with a coverage > 70% on larger genomes and coverage >90% on smaller genomes. Here, the coverage was calculated by merging the aligned fraction length of BLASTn high‐scoring pair sequences that shared at least 90% nucleotide similarity. Finally, a MCL^[^ ^34^ ^]^ (v14‐137) was used with an inflation value of 4.0, which took the filtered BLASTn results as input, carried out graph‐based clustering and clustered the viral contigs into VCs.

### Identification of crAssphages and Gubaphages in CHGV Contigs

crAss‐like phage genomes were annotated by following the method reported in a previous study.^[^ ^83^ ^]^ First, the nucleotide sequences of all CHGV contigs were subjected to search against the protein sequences of the polymerase (UGP_018) and the terminase (UGP_092) of the prototypical crAssphage (p‐crAssphage, NC_02 4711.1) using BLASTx. Second, the nucleotide sequence similarities between the CHGV contigs and the p‐crAssphage genome were assessed using BLASTn. A contig was then labeled as a putative crAssphage when it was longer than 70 kb and met at least one of the following criteria:

1. BLASTx hit with an E‐value <1e‐10 against either p‐crAssphage polymerase or terminase
2. ≥95% nucleotide identity over 80% of the contig length with the p‐crAssphage genome

Gubaphage genomes were annotated by clustering viral contigs with the Gubaphage genomes obtained from the GPD database^[^ ^2b^ ^]^ into VCs using the methods mentioned above. Viral contigs that were in the same VC as Gubaphage were annotated as Gubaphages.

### Estimation of The Prevalence of The CHGV vOTUs

To estimate the prevalence of viral contigs, the viral short‐read clean data were mapped to the CHGV database using Bowtie2. Then, the “presence” of a viral genome was defined in a sample if over 50% of its length was covered by the aligned reads from the sample with >4X overall sequencing depth.

### Estimation of The Relative Abundance of The CHGV vOTUs At The Viral Contig and VC Levels

To estimate the abundance of viral contigs in a sample, the viral short‐read clean reads were mapped to the CHGV database using Bowtie2. Then, the reads per kilobase million (RPKM) value of each viral contig. was calculated. Viral contigs were excluded that were not “present” in the sample (see the definition of the “presence” of a viral genome in a sample in the above section) and set their relative abundances to zero. Relative species abundance was calculated by dividing the RPKM of a specific viral contig by the total RPKM of all viral contigs that presented in the sample.

The relative abundance of a VC was calculated as the summation of the abundances of all its member viral contigs.

### Prediction of Viral Lifestyles

The lifestyle classifications of all the CHGV vOTUs were analyzed using DeePhage^[^ ^84^ ^]^ v1.0 with the default parameters. DeePhage uses a scoring system to classify viral genomes into four categories, including temperate (with scores ≤0.3), uncertain temperate (0.3–0.5), uncertain virulent (0.5–0.7), and virulent (>0.7). Higher scores indicate higher virulence. According to a benchmark study,^[^ ^85^ ^]^ DeePhage can classify short contigs from metagenomic data and has the best reported performance on lifestyle prediction, while BACPHLIP^[^ ^86^ ^]^ was designed for complete phage genomes. And DeePhage has better generalization ability on novel phages as using a deep neural network to learn features from both DNA and protein sequences of phages, while BACPHLIP relies on a set of conserved protein domains that were associated with lysogeny.

### Prediction of Virus‐Host Relationships

Virus‐host relationships were predicted based on two methods. First, CRISPR (Clustered Regularly Interspaced Short Palindromic Repeats)‐prokaryote relationships. A catalog of such relationships was compiled from the following sources, including 1) spacers from CRISPR Spacers Database and their host information,^[^ ^87^ ^]^ 2) spacers predicted from the genomes in the NCBI prokaryotic RefSeq database^[^ ^88^ ^]^ (14649 genomes, as of Sep 2021), 3) spacers predicted from the UHGG genomes using Piler‐CR v1.06.^[^ ^89^ ^]^ Spacer‐host relationships were also predicted using the mNGS data. Briefly, the mNGS data were assembled using IDBA‐UD v1.1.3,^[^ ^61^ ^]^ followed by spacer prediction on the assembled contigs using CRISPRCasFinder v4.2.20.^[^ ^90^ ^]^ The taxonomic classification of the contigs was predicted using GTDB‐TK.^[^ ^91^ ^]^ Spacers shorter than 20 bp were considered as low confidence. Spacers were aligned to the HGV database using Bowtie2 to identify the putative phage‐host interactions.

Second, tRNA gene‐prokaryote relationships. Prokaryotic tRNAs could be incorporated into viruses during viral assembly and packaging; such a relationship could be used to establish virus‐host relationships.^[^ ^2f^ ^]^ Viral tRNA genes and bacterial tRNA genes were predicted using tRNAscan‐SE v2.0.9^[^ ^92^ ^]^ (http://lowelab.ucsc.edu/tRNAscan‐SE/) on all the above‐mentioned genomes and assembled contigs. Bacterial tRNA genes that matched viral tRNA genes at 95% identity across 100% of the length were used for establishing virus‐bacterial host relationships.

Virus‐host relationships supported by multiple evidence (i.e., > = 2 spacers, > = 2 tRNAs, or > = 1 spacers and > = 1 tRNAs) were considered as high confident; others were considered as of low confidence. Host range was calculated for all the VPs and VCs. For those with multiple predicted hosts, the last common ancestor (LCA) of all the hosts on the NCBI taxonomic database was calculated using an in‐house R script. The virus‐host relationships can be found in Table S7 (Supporting Information).

The association network between gut phages and their hosts was visualized using Cytoscape v3.8.2.^[^ ^43^ ^]^

### Statistics and Other Bioinformatics Analyses

All processed data, if not otherwise stated, were loaded into R (v4.0.5, https://www.r‐project.org/), analyzed or visualized.

### Ethics Approval

This study was approved by the Ethics Committee of Tongji Medical College of Huazhong University of Science and Technology (No, S1241) and the Human Ethics Committee of the School of Life Sciences of Fudan University (No, BE1940).

## Conflict of Interest

The authors declare no conflict of interest.

## Author Contributions

J.C., C.S., Y.D., and M.J. contributed equally to this work. W.H.C., X.M.Z., Z.L., and P.B. designed and directed the research; J.C. managed the sampling and performed most of the experiments; C.S. performed most of the analysis; Y.D. and H.W. helped with the analysis. X.Z. and M.J. also helped with the sample collection and phage enrichment experiments; C.S. and J.C. wrote the paper with results from all authors; W.H.C., X.M.Z., Z.L., and P.B. polished the manuscript through multiple iterations of discussions with all authors. All authors have read and approved the final manuscript.

## Supporting information

Supporting Information

Supplementary Table 1

Supplementary Table 2

Supplementary Table 3

Supplementary Table 4

Supplementary Table 5

Supplementary Table 6

Supplementary Table 7

Supplementary Table 8

Supplementary Table 9

## Data Availability

The raw sequencing data used in this study are available in the CNCB GSA database under accession code PRJCA007087 (https://ngdc.cncb.ac.cn/gsa/s/jj9JJ2DO, for review only), and the NCBI SRA database under accession code PRJNA835720. The viral sequence datasets generated by this study and related meta‐data have been uploaded to a Figshare repository at https://figshare.com/projects/Efficient_recovery_of_complete_gut_phage_genomes_by_combined_short‐_and_long‐read_sequencing/155306.
