# Supplementary material for: Efficient Recovery of Complete Gut Viral Genomes by Combined Short‐ and Long‐Read Sequencing
Source: Adv Sci (Weinh). 2024 Jan 19;11(13):2305818. doi: 10.1002/advs.202305818 (PMC10987132; doi:10.1002/advs.202305818)
Supplement: Supplementary file 1 — Supporting Information [file ADVS-11-2305818-s010.pdf]

ADVANCED SCIENCE
Open Access

Supporting Information

for *Adv. Sci.*, DOI 10.1002/advs.202305818

Efficient Recovery of Complete Gut Viral Genomes by Combined Short- and Long-Read Sequencing

Jingchao Chen, Chuqing Sun, Yanqi Dong, Menglu Jin, Senying Lai, Longhao Jia, Xueyang Zhao, Huarui Wang, Na L. Gao, Peer Bork, Zhi Liu, Wei-Hua Chen* and Xing-Ming Zhao**

Supporting Tables

Table S1. Detailed meta information of subjected samples.
Table S2. A list of 21,499 vOTUs and related information in details.
Table S3. Validation of highly prevalent vOTUs by Models trained on various public gut virus datasets.
Table S4. Information of highly prevalent vOTUs across public datasets.
Table S5. Detailed information about highly prevalent vOTUs in CHGV.
Table S6. Host information of highly prevalent vOTUs annoted by Hophage and PHIAF.
Table S7. A list of gene function groups and the number of genes they contain.
Table S8. Host information of the vOTUs with host assignment.
Table S9. A list of 21,499 viral genomes and their assigned 9,432 bins.

Supporting Figures

Figure S1

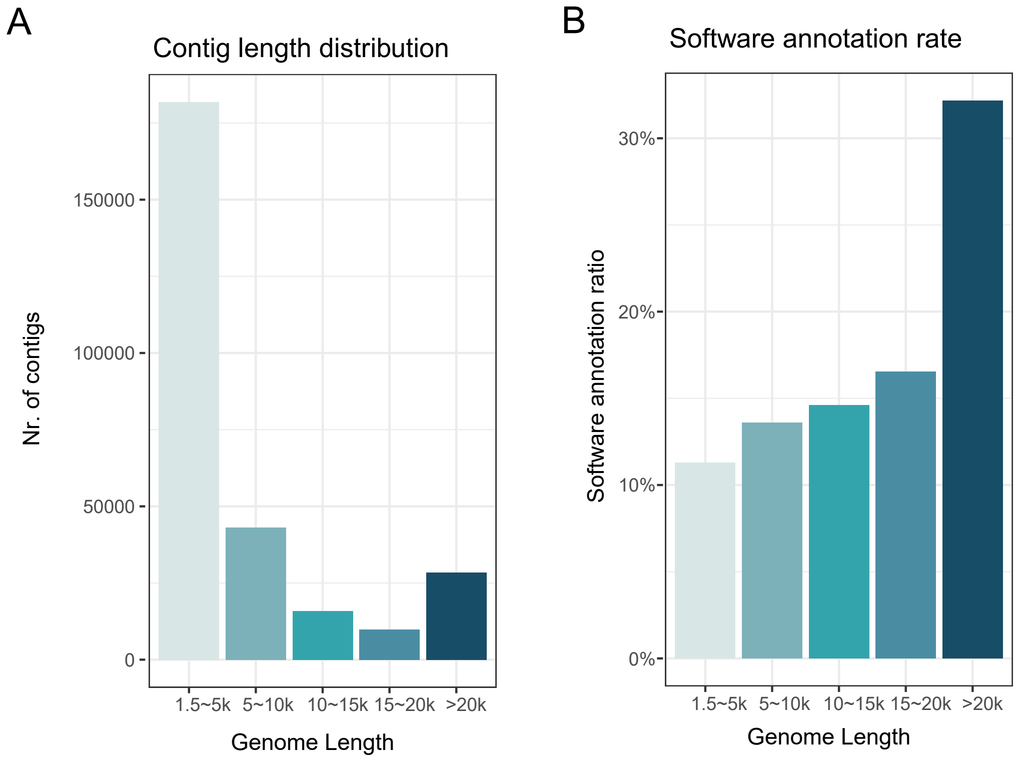

Figure S1, A—B, bar plot showing the count and software annotation rate of CHGV vOTUs in different length range. The longer the vOTU is, the more likely it is to be recognized by virus detection software.

Figure S2

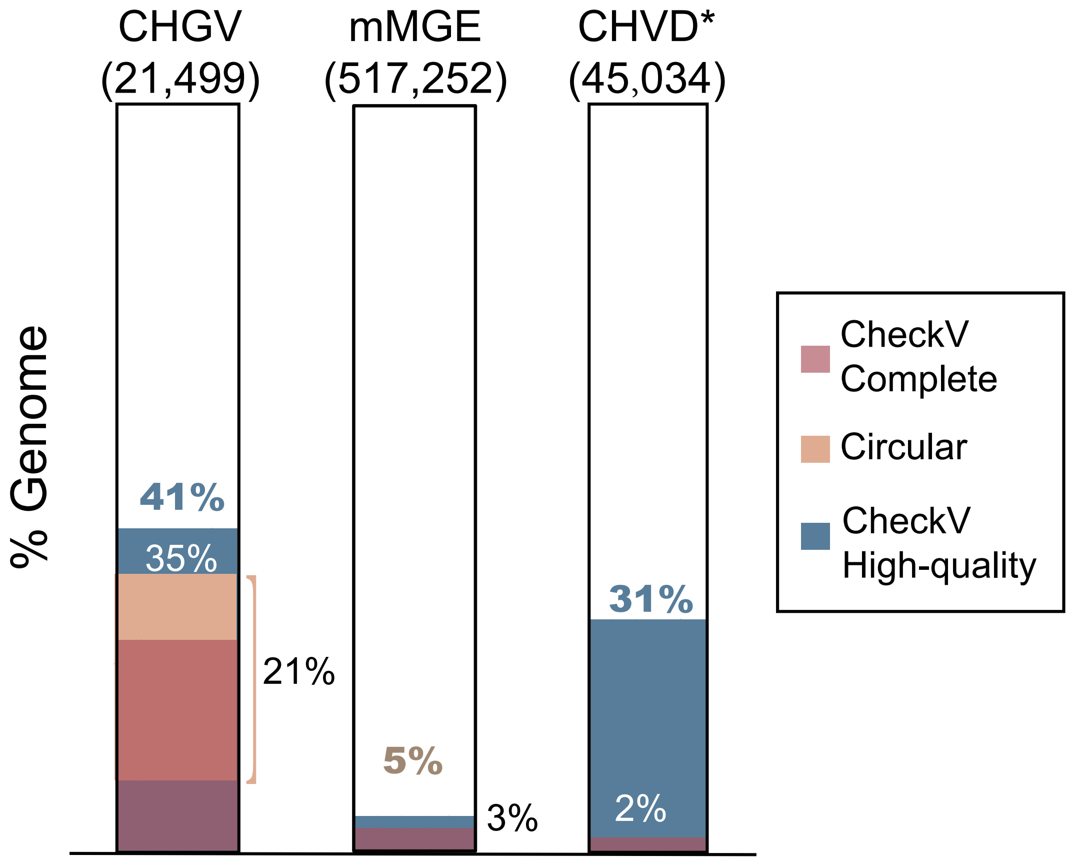

Figure S2, A—B, Bar plot comparing the complete genome ratio among databases (Dark red: CheckV completeness 100%, ∼28%; light pink: circular , ∼21%; Dark blue: CheckV high-quality, ∼41%). mMGV: The human metagenomic extrachromosomal mobile genetic elements database. GHVD: The Cenote human virome database.

Figure S3

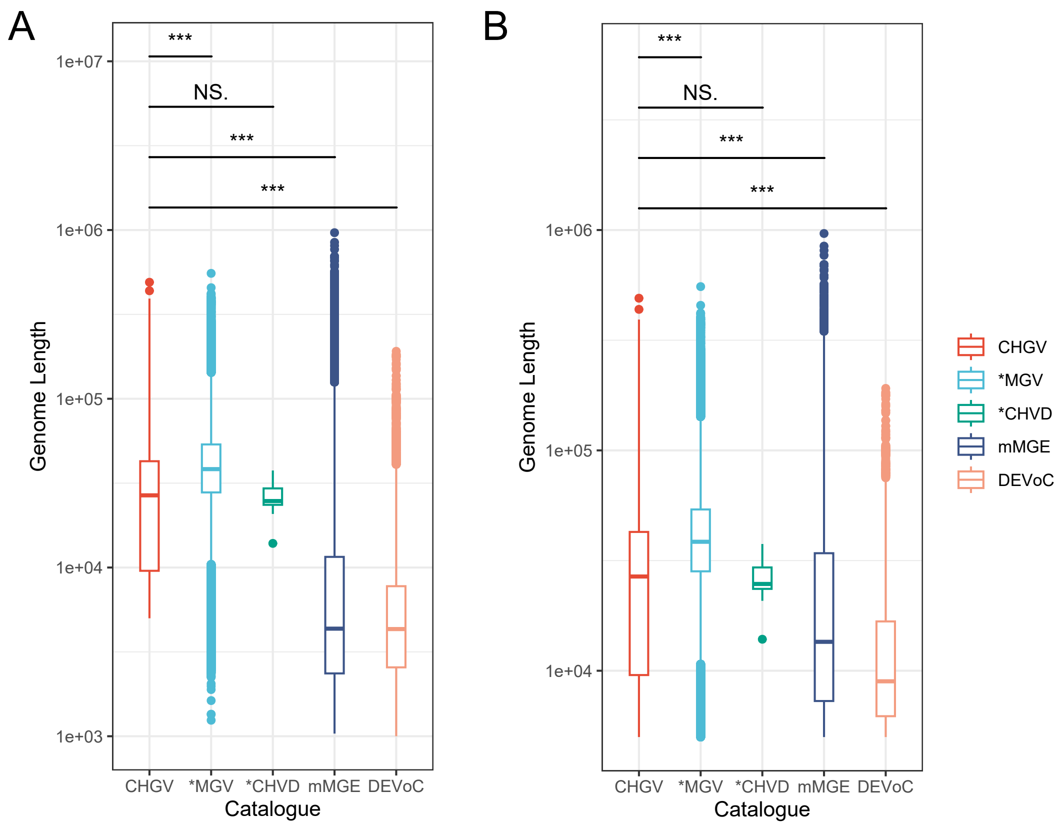

Figure S3, A—B, Genome length comparisons of the all vOTUs and vOTUs greater than 5kb in selected public viral catalogues.

Figure S4

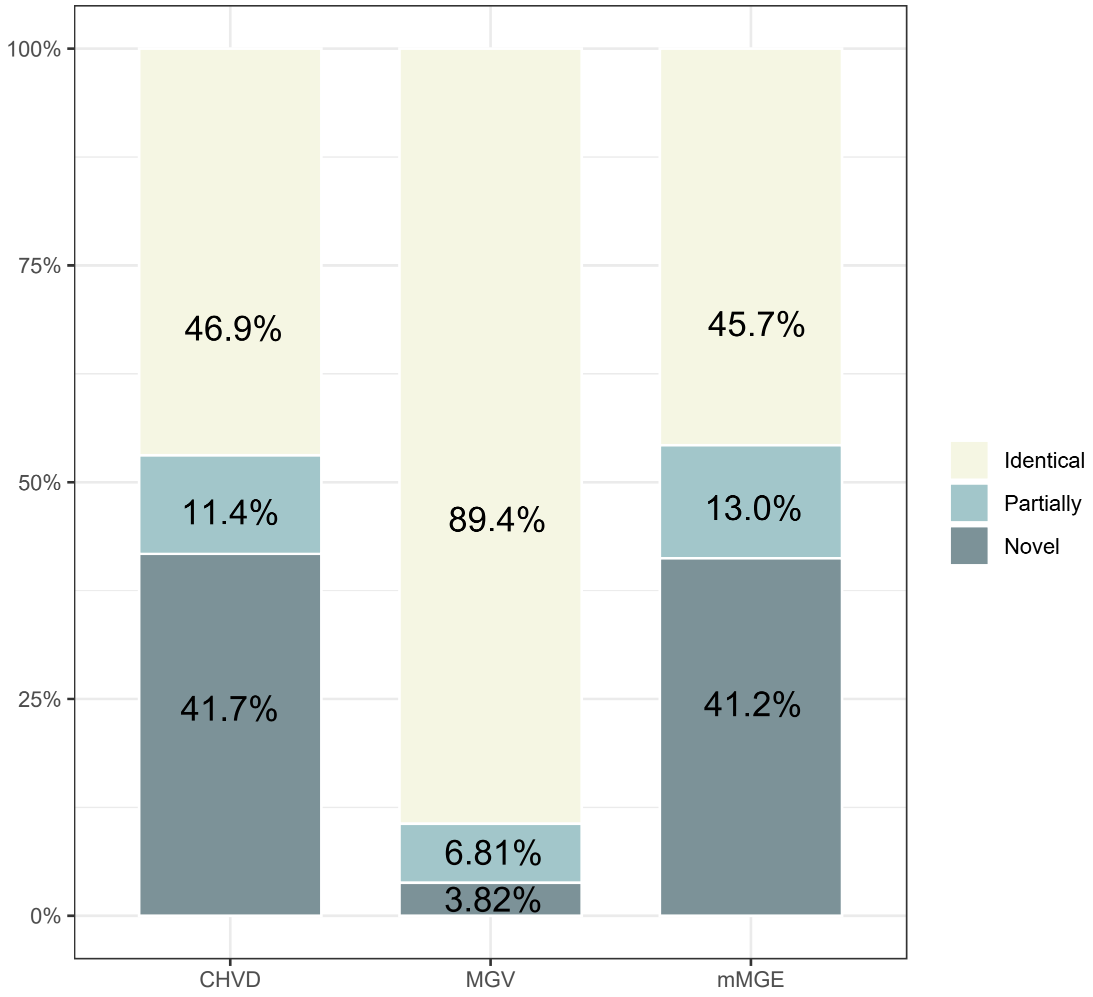

Figure S4, Bar plot showing the novelty of the CHGV and selected public human viral catalogues as compared with all other human viral catalogues including CHVD, MGV and mMGE.

Figure S5

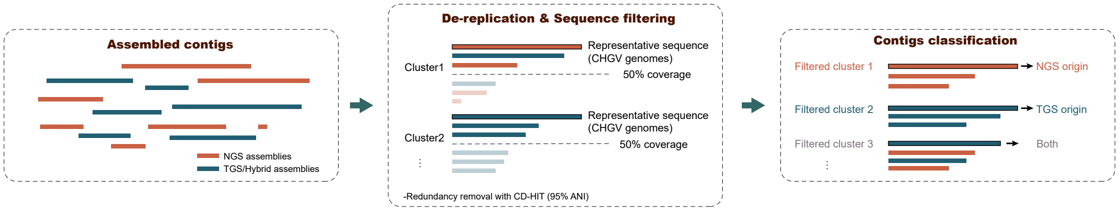

Figure S5, Diagram showing the origin classification of the CHGV vOTUs.

Figure S6

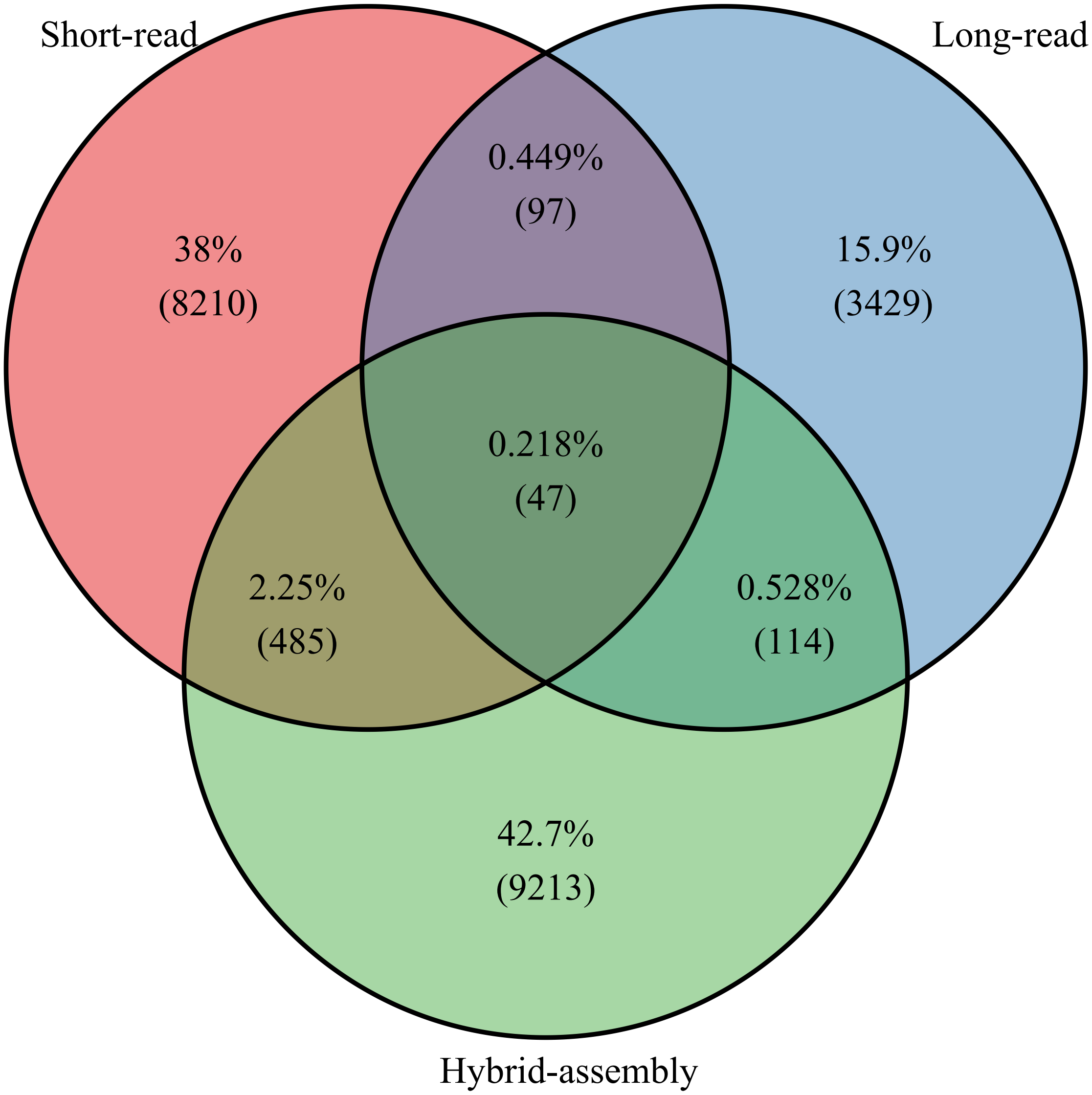

Figure S6, Venn plot showing the number of vOTUs generated by different assembly methods.

Figure S7

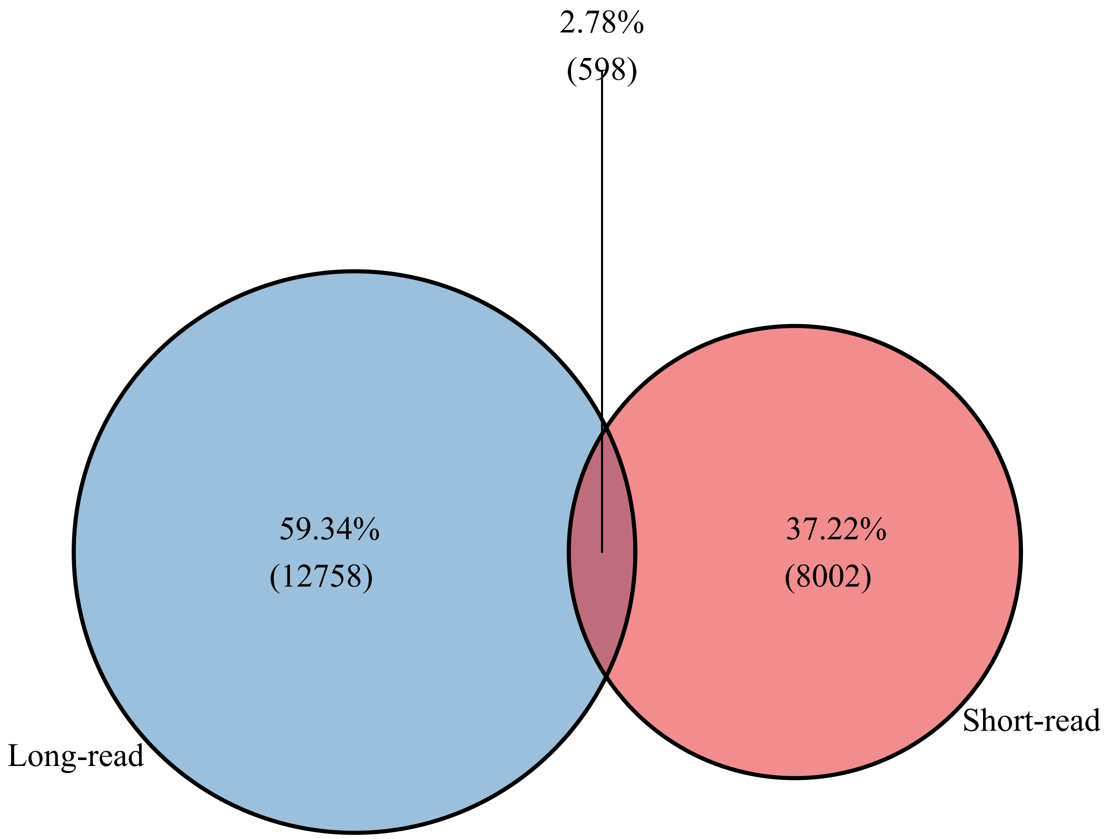

Figure S7, Venn plot showing the contributions of the long- and short-reads to the CHGV viral genomes limiting the samples to those with both short-reads and long-reads sequencing data

Figure S8

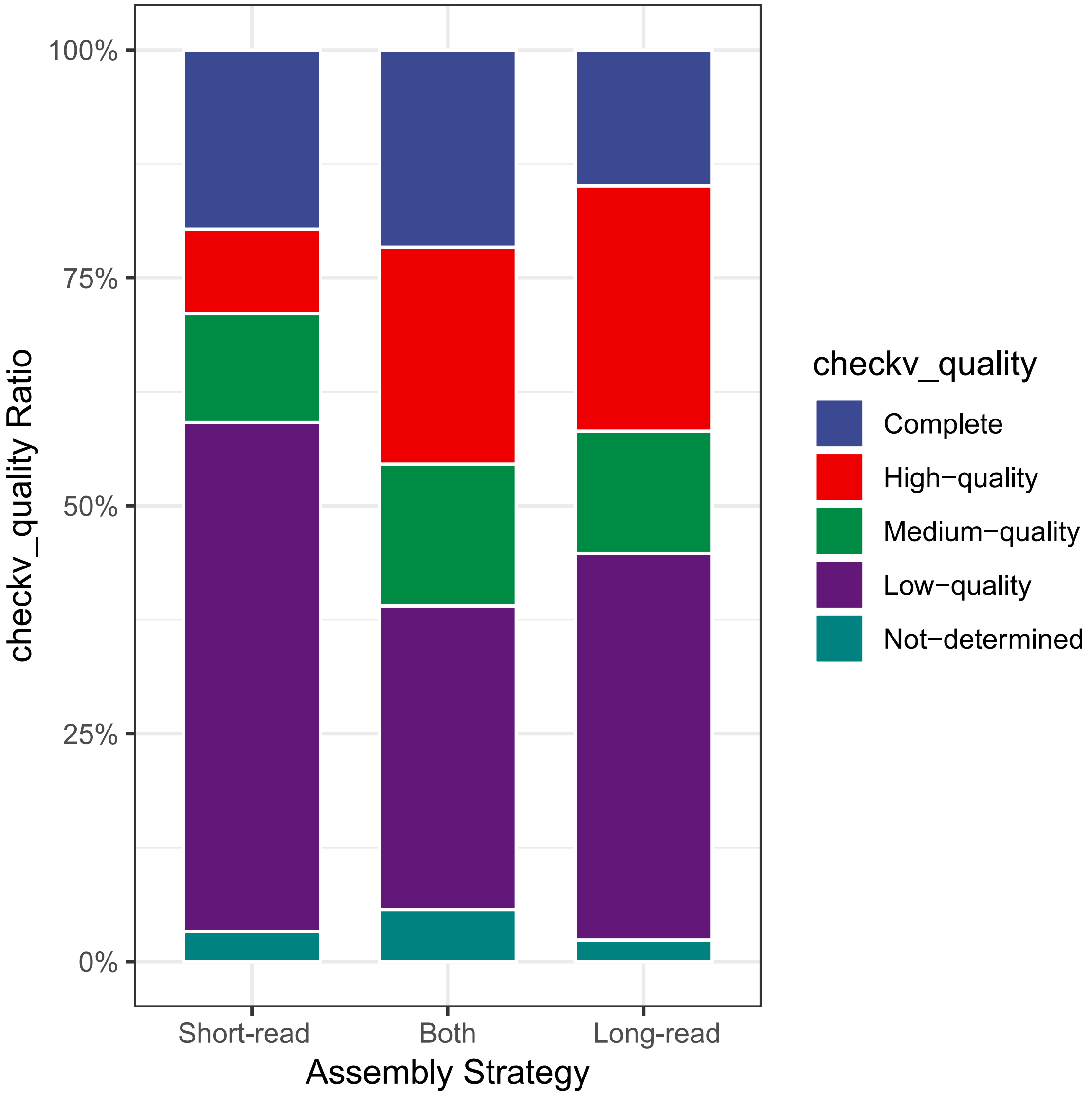

Figure S8, Stack bar plot showing the genome quality of genomes with different origins.

Figure S9

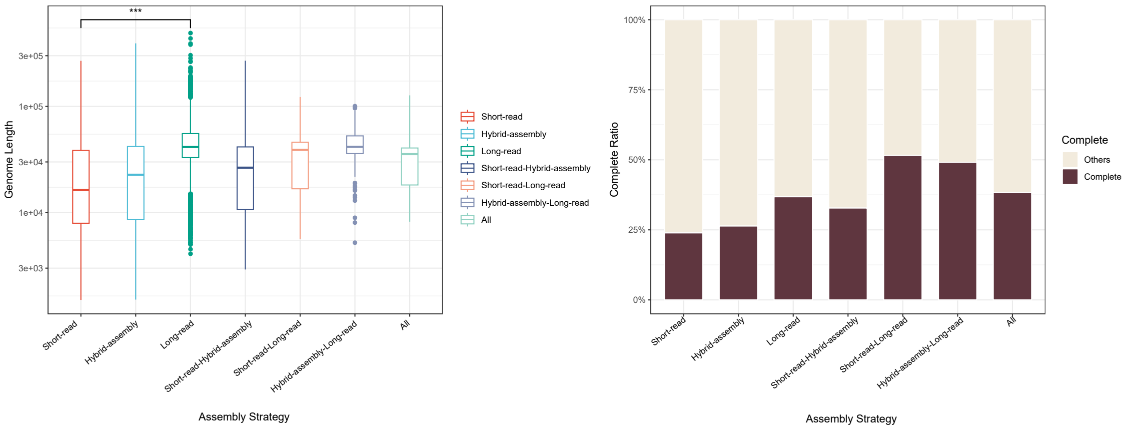

Figure S9, Genome length comparisons of the vOTUs obtained by various assembly strategies.

Figure S10

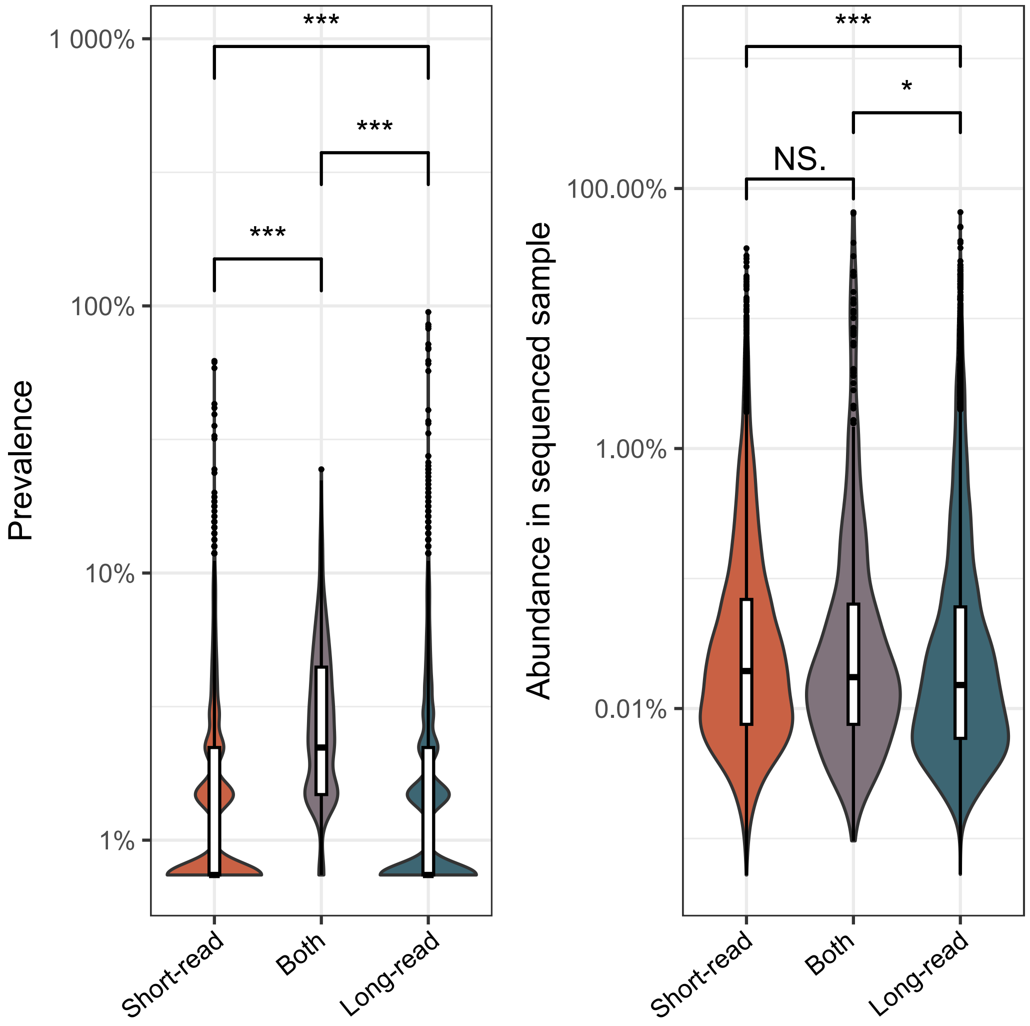

Figure S10, Violin plots showing the prevalence and abundance of sequence sample of genomes with different origins.

Figure S11

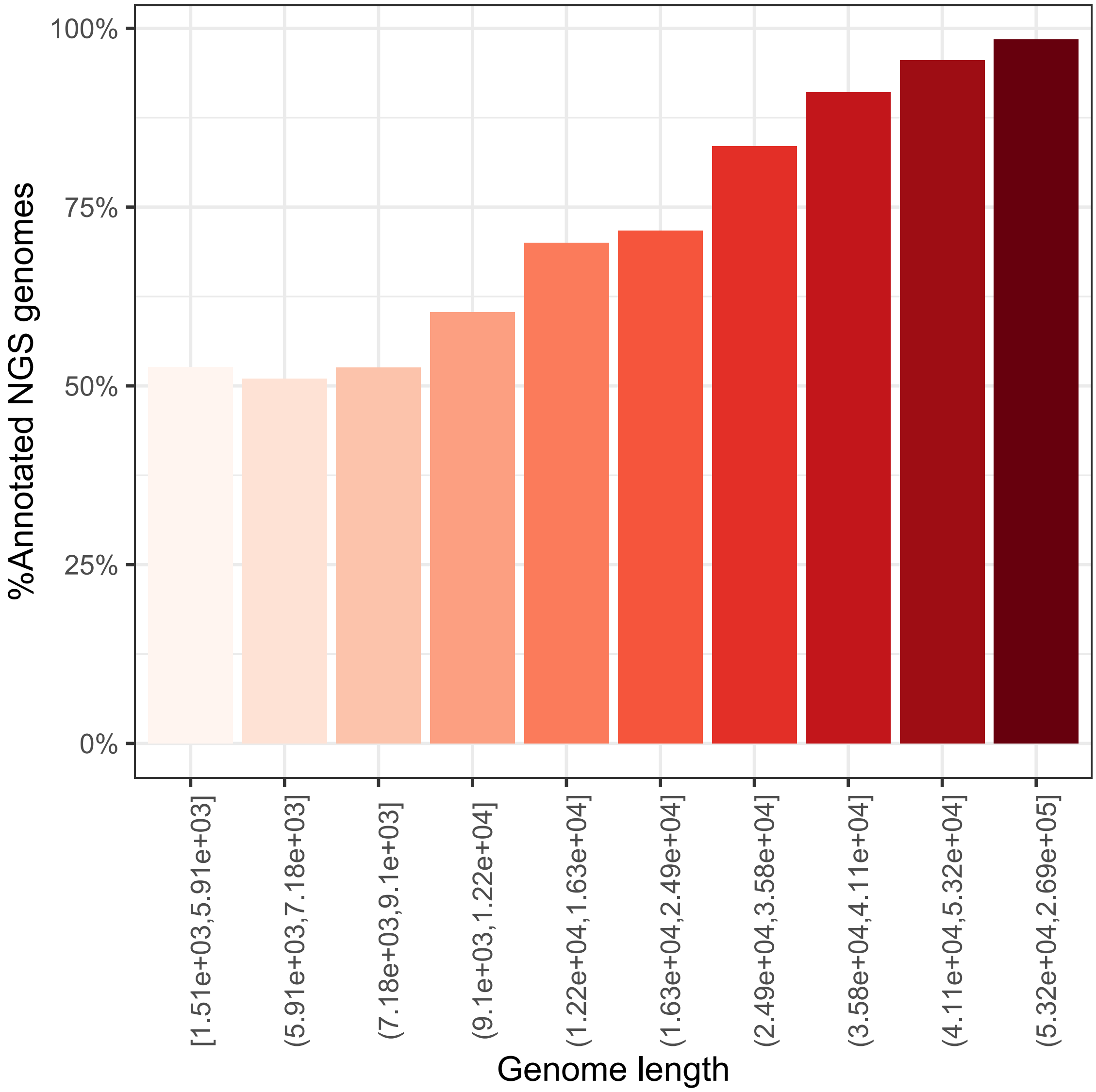

Figure S11, Bar plot showing the annotation ratio of short-read vOTUs in different length ranges.

Figure S12

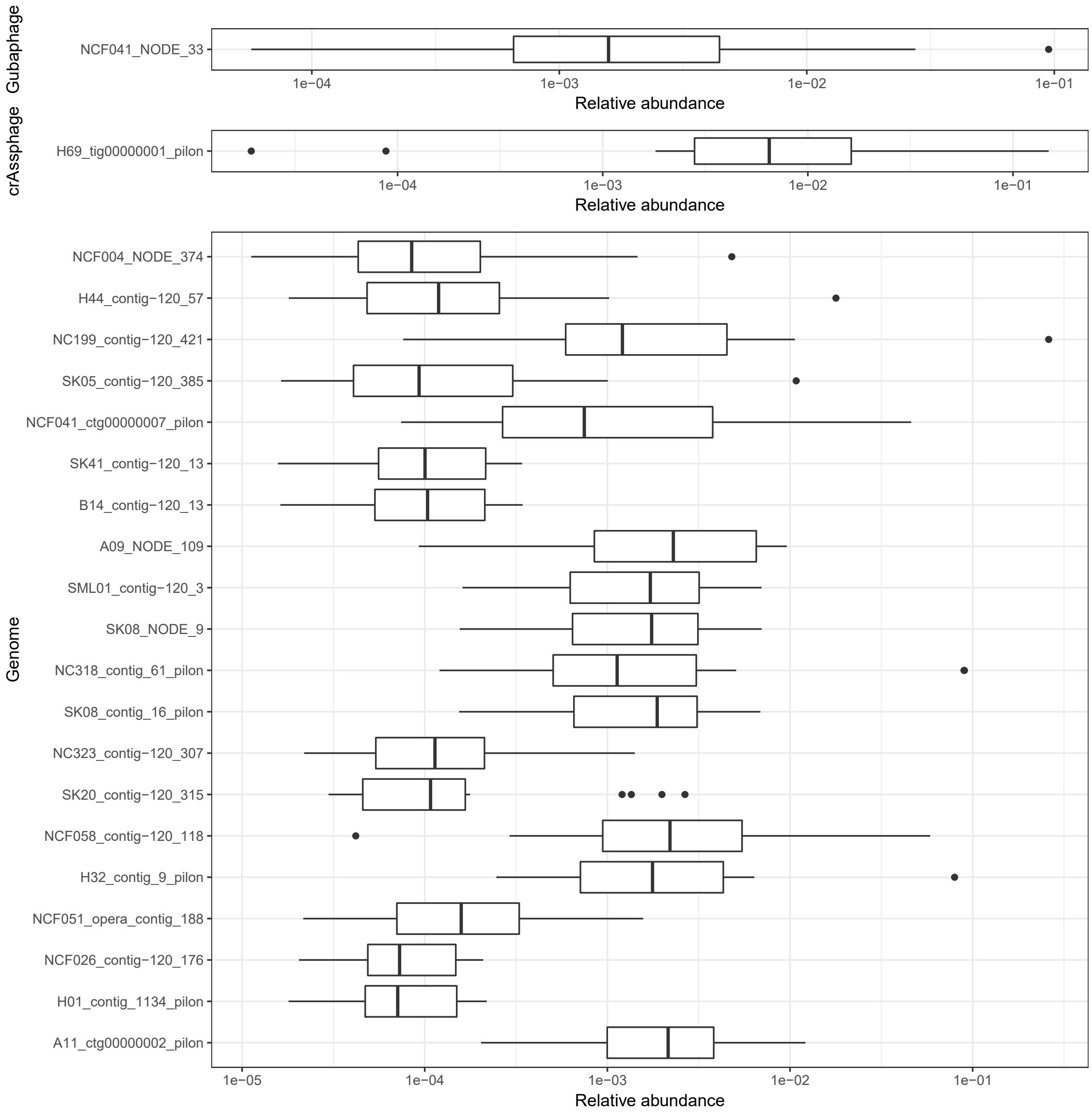

Figure S12, Boxplots showing the abundance distribution of selected high-prevalence vOTUs.

Figure S13

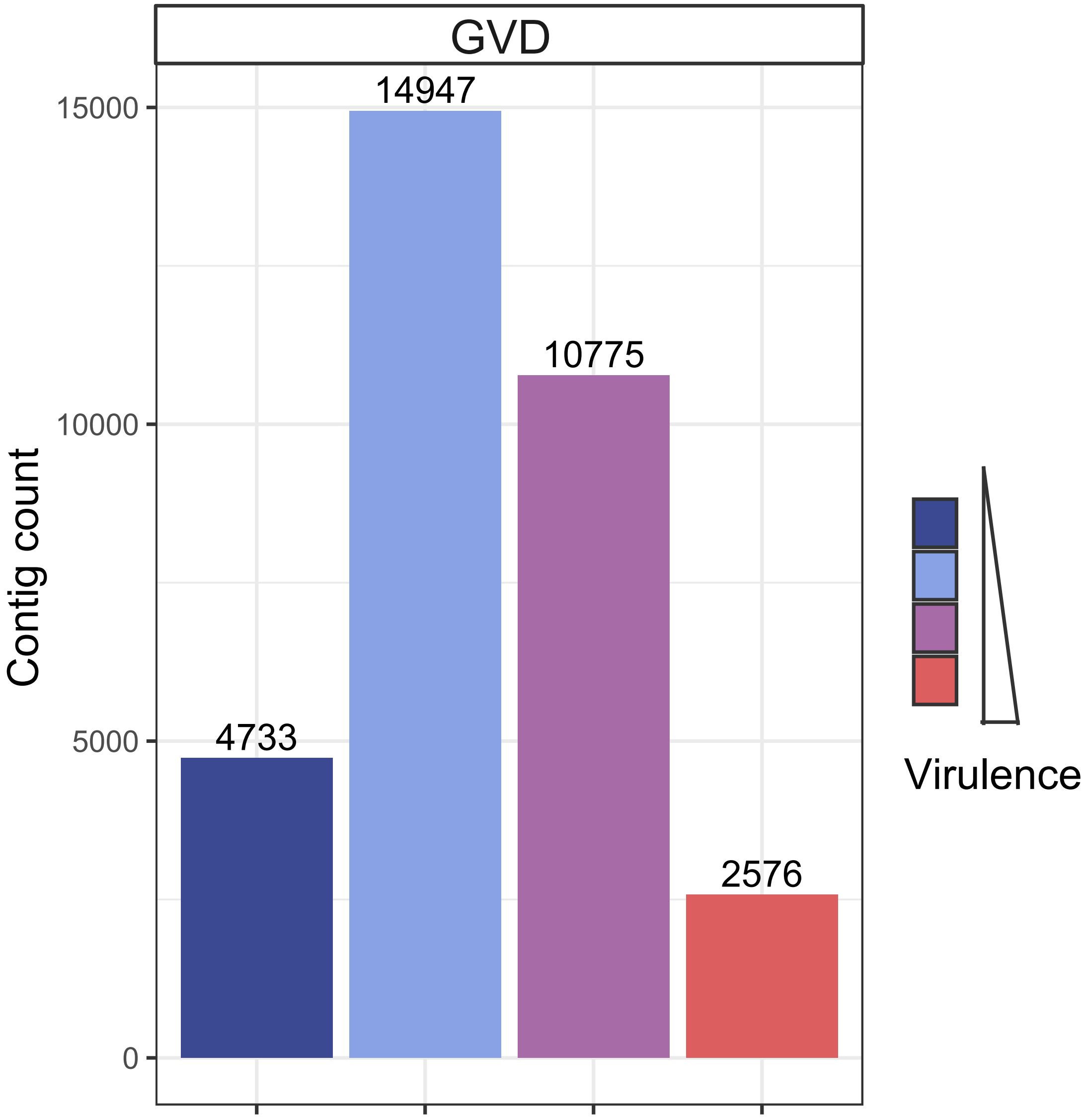

Figure S13, GVD contigs' lifestyle. The temperate and uncertain temperate vOTUs accounted of 68% of GVD vOTUs.

Figure S14

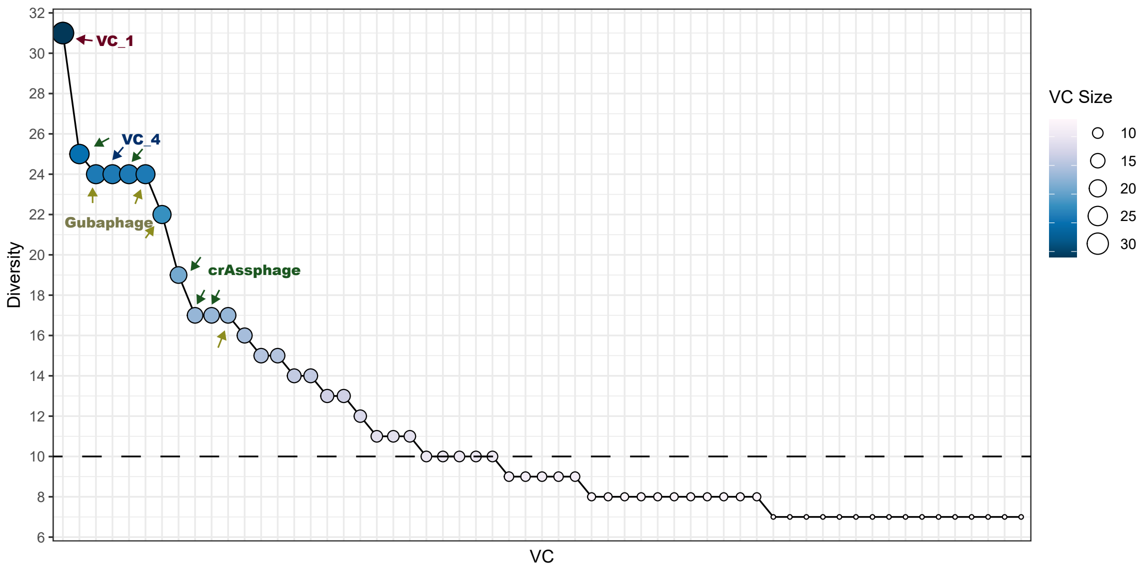

Figure S14, Top VCs ranked by their diversities (i.e., numbers of included viral populations (VPs), VC size). Each dot represents a VC. Colored arrows pointed to different types of Bacteriophages, such as crAssphages and Gubaphages (green and yellow respectively).

Figure S15

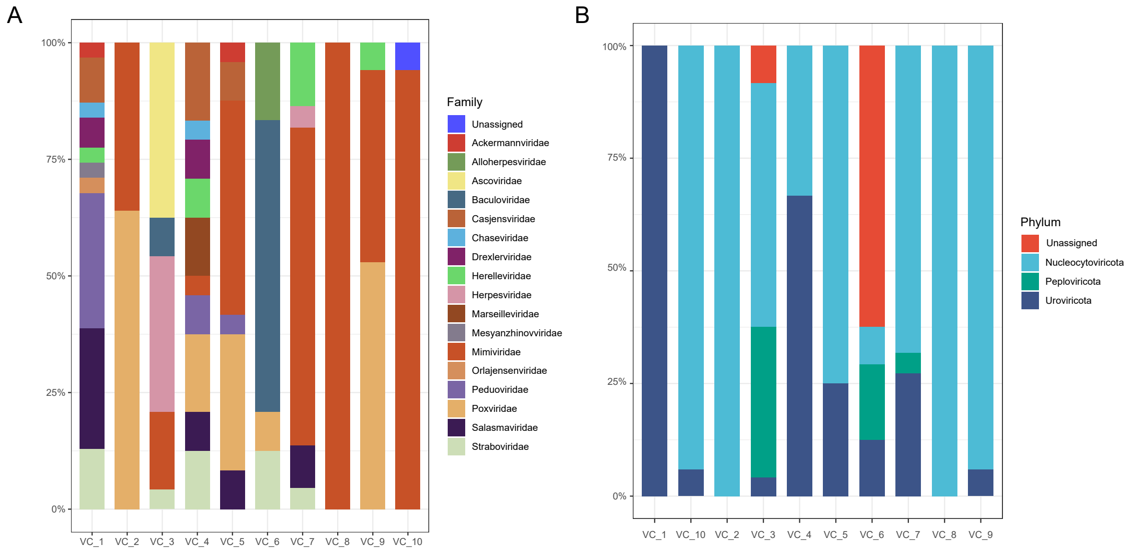

Figure S15, Taxonomic annotation of the top 10 VCs at Family and phylum level.

Figure S16

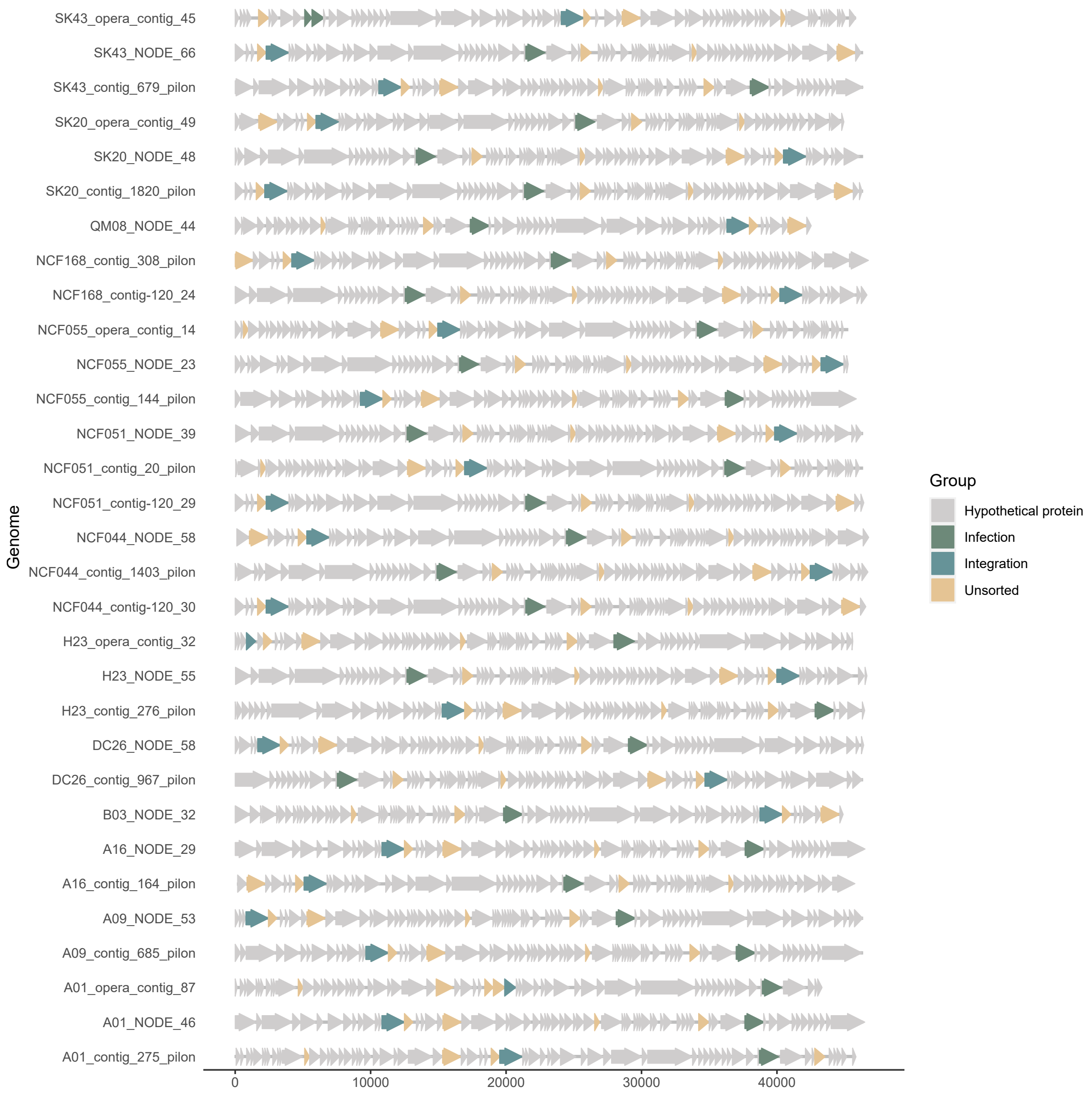

Figure S16, Genomes annotation of vOTUs in VC1. The line-arrow charts show the genome annotation results of virus in the corresponding viruses. The annotated protein-coding genes (arrows) are colored according to their viral function, including lysis, infection and integration (Methods).

Figure S17

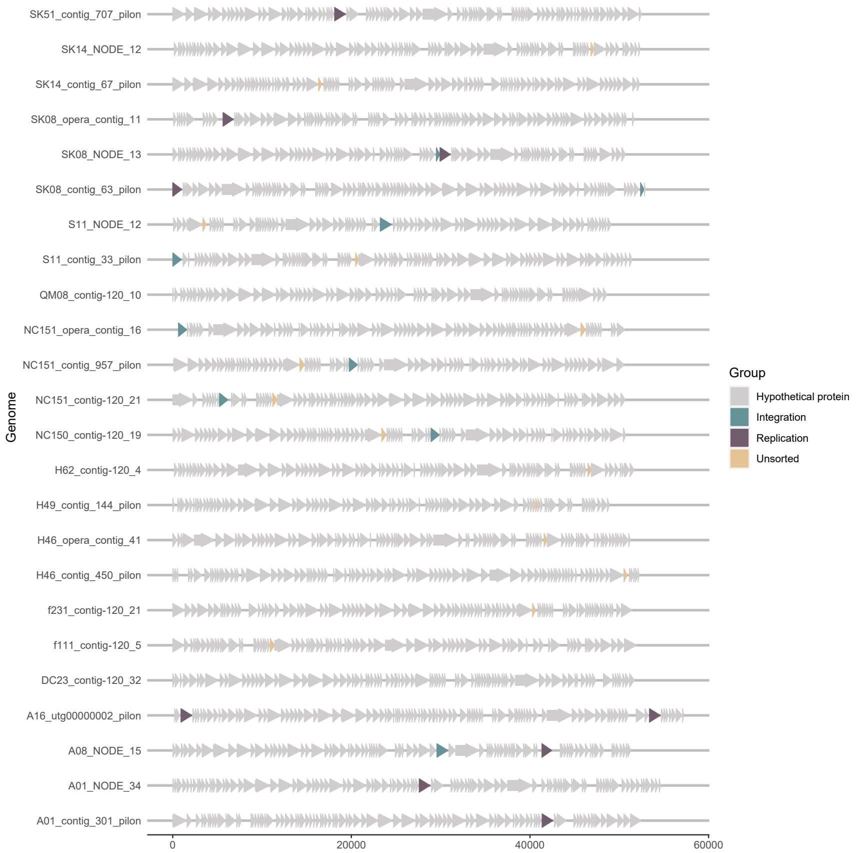

Figure S17, Genomes annotation of vOTUs in VC4. The line-arrow charts show the genome annotation results of virus in the corresponding viruses. The annotated protein-coding genes (arrows) are colored according to their viral function, including lysis, infection and integration (Methods).

Figure S18

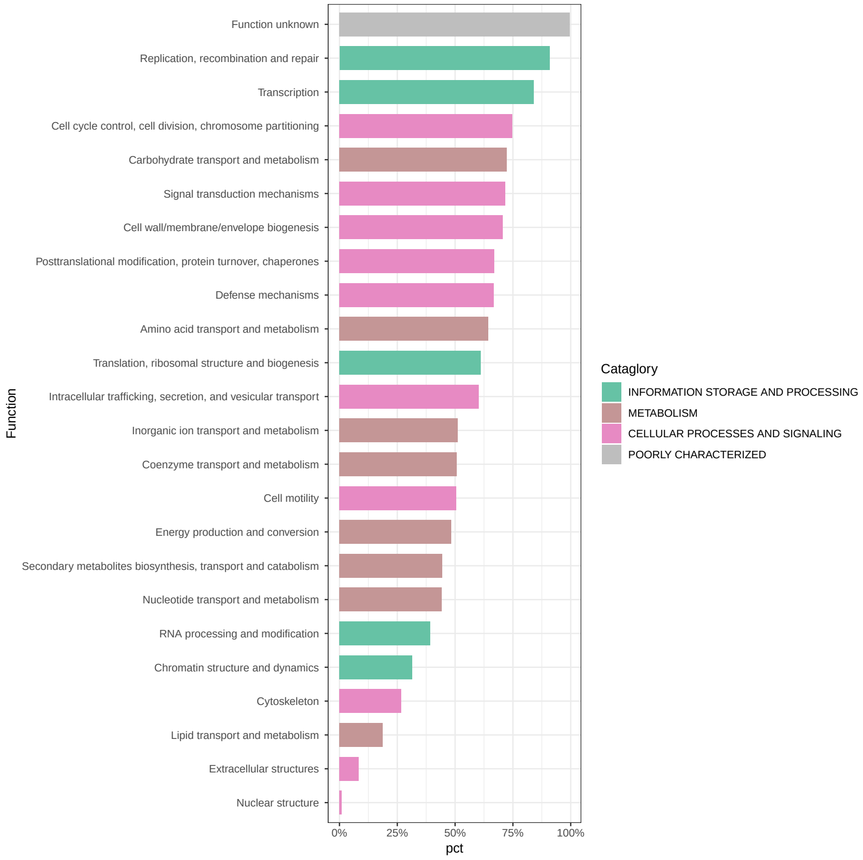

Figure S18, Bar plot showing the prevalence of each function group.

Figure S19

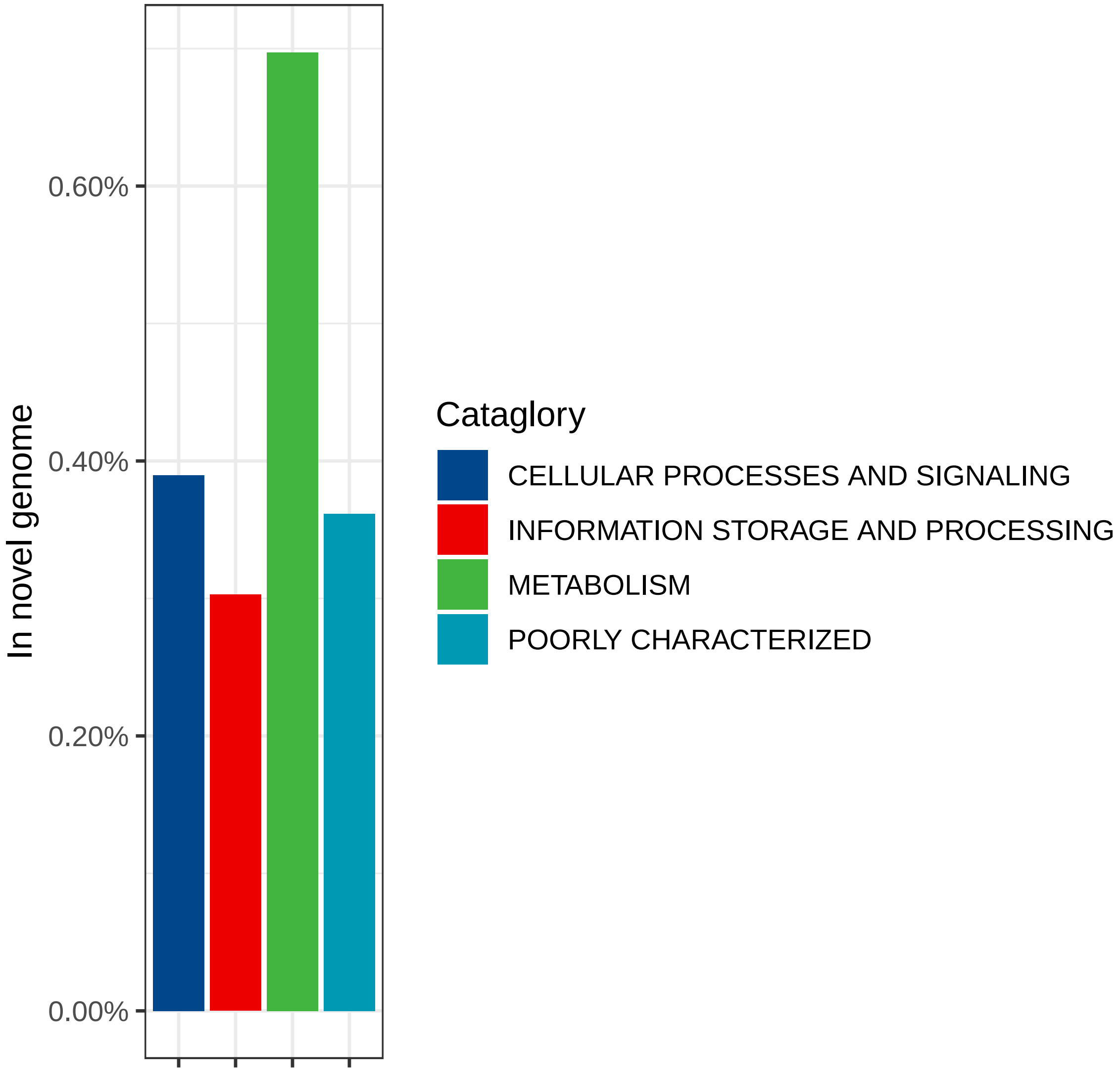

Figure S19, Bar plot showing ratio of proteins with novel functions in novel vOTUs.

Figure S20

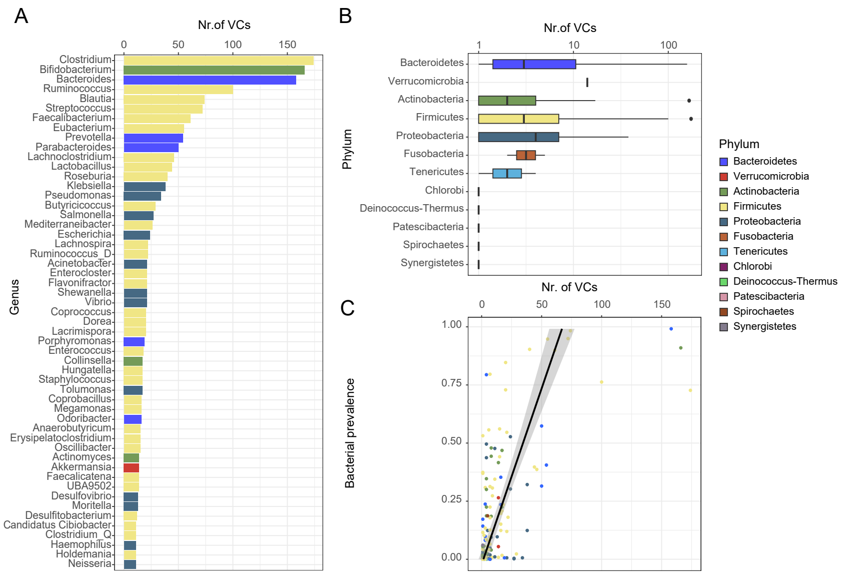

Figure S20, A, Bar plot showing the top 50 genus with the highest number of VCs. **B,** Boxplot showing the number of VCs each genus contains of different phyla. **C,** The correlation of bacterial prevalence and the number of VCs it consists of. More prevalent bacterial clades were associated with more VP and VCs

Figure S21

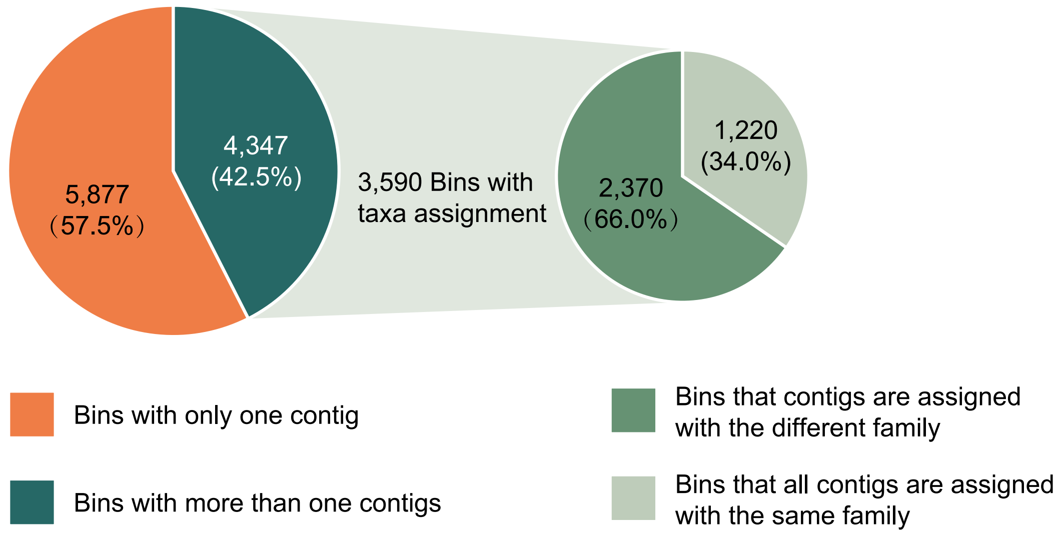

Figure S21, Pie charts displaying the percentage of VAMB singleton bins vs. non-singleton bins and whether the contigs in non-singleton bins were assigned with the same family.
